# Origin of an Assemblage Massively Dominated by Carnivorans from the Miocene of Spain

**DOI:** 10.1371/journal.pone.0063046

**Published:** 2013-05-01

**Authors:** M. Soledad Domingo, M. Teresa Alberdi, Beatriz Azanza, Pablo G. Silva, Jorge Morales

**Affiliations:** 1 Museum of Paleontology, University of Michigan, Ann Arbor, Michigan, United States of America; 2 Departamento de Paleobiología, Museo Nacional de Ciencias Naturales-Consejo Superior de Investigaciones Científicas, Madrid, Spain; 3 Departamento de Ciencias de la Tierra, Facultad Ciencias, Instituto Universitario de Investigación en Ciencias Ambientales de Aragón, Universidad de Zaragoza, Zaragoza, Spain; 4 Departamento de Geología, Universidad de Salamanca, Escuela Politécnica Superior de Ávila, Ávila, Spain; Raymond M. Alf Museum of Paleontology, United States of America

## Abstract

Carnivoran-dominated fossil sites provide precious insights into the diversity and ecology of species rarely recovered in the fossil record. The lower level assemblage of Batallones-1 fossil site (Late Miocene; Madrid Basin, Spain) has yielded one of the most abundant and diversified carnivoran assemblage ever known from the Cenozoic record of mammals. A comprehensive taphonomic study is carried out here in order to constrain the concentration mode of this remarkable assemblage. Another distinctive feature of Batallones-1 is that the accumulation of carnivoran remains took place in the context of a geomorphological landform (cavity formation through a piping process) practically unknown in the generation of fossil sites. Two characteristics of the assemblage highly restrict the probable causes for the accumulation of the remains: (1) the overwhelming number of carnivorans individuals; and (2) the mortality profiles estimated for the four most abundant taxa do not correspond to the classic mortality types but rather were the consequence of the behavior of the taxa. This evidence together with other taphonomic data supports the hypothesis that carnivoran individuals actively entered the cavity searching for resources (food or water) and were unable to exit. The scarcity of herbivores implies that the shaft was well visible and avoided by these taxa. Fossil bones exhibit a very good preservation state as a consequence of their deposition in the restricted and protective environment of the chamber. Batallones-1 had another assemblage (upper level assemblage) that was dominated by herbivore remains and that potentially corresponded to the final stages of the cavity filling.

## Introduction

Mammalian carnivores constitute a small portion of the biomass in modern ecosystems as a result of their position at the top of the trophic pyramid; specifically, the herbivore to carnivore ratio has been estimated in 10∶1 [1], [2]. This pattern is generally reflected in the fossil record so that herbivore remains commonly outnumber carnivoran remains in mammalian fossil localities. Klein and Cruz Uribe [3] estimated that carnivorans are usually represented by less than 10% of the total Number of Identified Specimens (NISP) and Minimum Number of Individuals (MNI) in mammalian fossil localities. Accordingly, carnivoran-rich fossil sites constitute unique windows to investigate the taxonomy and ecology of these animals in the past. Additionally, these fossil sites provide precious information on the diversity of carnivorans in the past. For example, in the case of the Spanish mammalian record, almost one third (∼30%) of the carnivoran species known for the MN10 unit (∼9.8 to 8.8 Ma; based on the MN biochronological framework for the mammalian fossil sites in Europe; [4]) are species only known from Cerro de los Batallones localities (Batallones Butte; Madrid Basin, Spain), the site area under study in this work.

Only a few carnivoran-dominated fossil sites are known in the fossil record, with the Pleistocene locality of Rancho La Brea as the best-known example. In Batallones-1 (BAT-1), the site studied here, carnivoran remains are even more abundant than in Rancho La Brea (98% in BAT-1 vs 85% in Pit 91 of Rancho La Brea as measured by NISP; [5], [6]). BAT-1 carnivoran remains belong to ten different Late Miocene taxa, that were previously unknown or poorly known in the fossil record, and include two species of sabertoothed cats, two felines, an amphicyonid, a hyaenid, an ailurid, a mustelid, and two mephitids [7]–[15]. This uncommon assemblage is allowing us to investigate carnivoran guilds from perspectives that are rarely permitted in the fossil record (e.g., predator-prey relationships through stable isotope analyses [16]).

**Figure 1:**
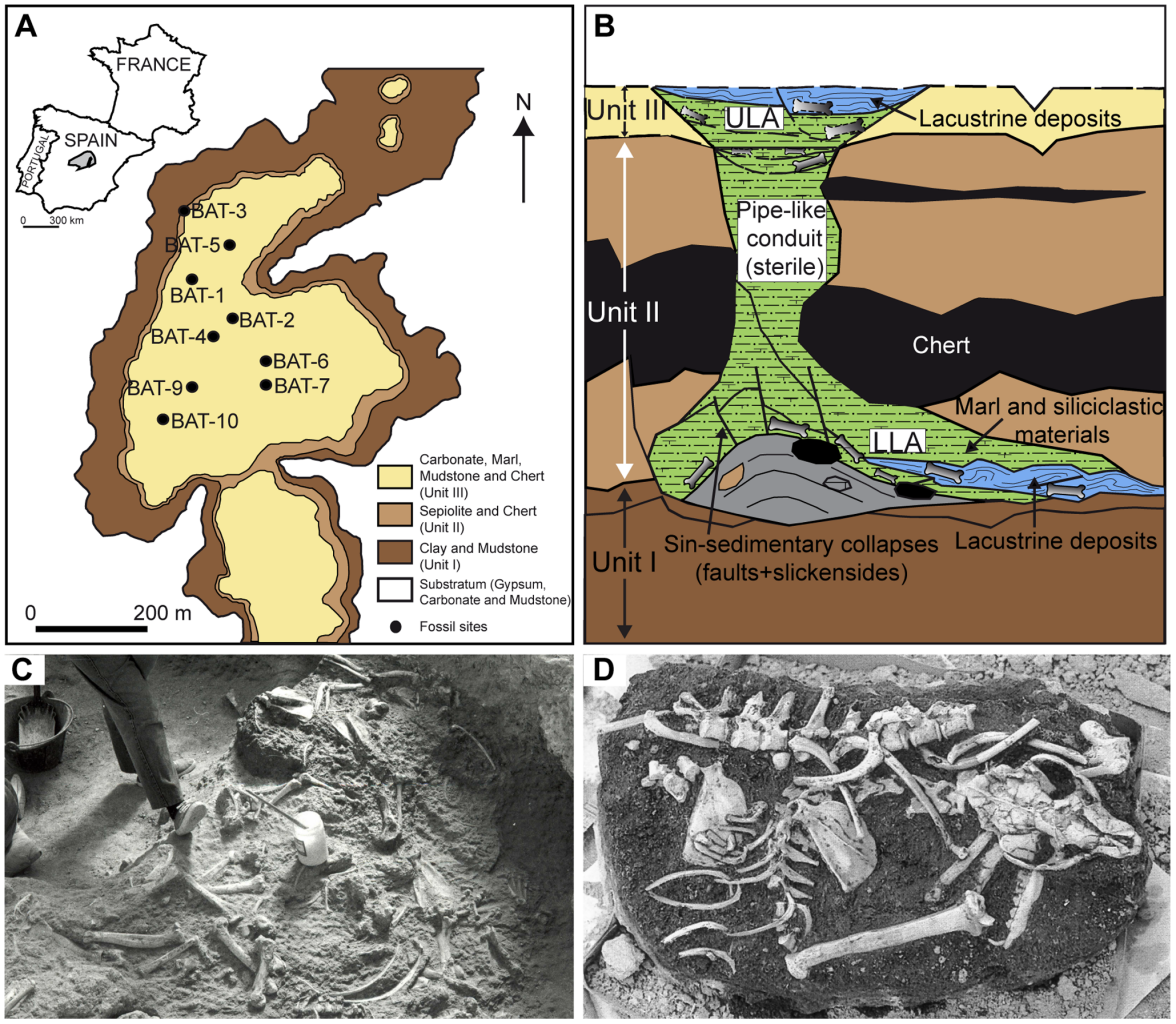
BAT-1 geographic, geologic and taphonomic context. A, Distribution of the three sedimentological units and fossil sites of Cerro de los Batallones in aerial view (Modified from Pozo et al. [17]). BAT-1 = Batallones-1, BAT-2 = Batallones-2 and so on. The Madrid Basin is highlighted in grey in the map of Spain; B, Geological sketch (vertical section) of BAT-1 showing the lower level assemblage (LLA) and upper level assemblage (ULA) (Modified from Pozo et al. [17]). Because the ULA was mostly eliminated during the sepiolite quarrying, we provide a reconstruction (dotted lines) based on observations from other localities of Cerro de los Batallones. The sketch is not to scale; C, BAT-1 LLA bone concentration. In some areas of the excavation, bones appeared tightly packed and in contact with one another; D, Partial skeleton of the hyaena *Protictitherium crassum*. Mean skull length of *Protictitherium crassum* from BAT-1 LLA is 13.5 cm.

BAT-1 was discovered in 1991 in the course of the quarrying of sepiolite clays in Cerro de los Batallones. More than 18,000 fossils were unearthed between that year and the last field season in 2008 (Fig. 1 and Fig. S1). Apart from BAT-1, eight more fossil localities have been discovered in the butte. All of them have a Late Vallesian age (∼10-9 Ma; early Late Miocene; [19], [20]). Two of the fossil sites (lower level of BAT-1 and BAT-3) exhibit taphocoenoses dominated by carnivoran remains, and practically all of the Cerro de los Batallones localities share taphonomic features such as an extremely high abundance and diversity of skeletal elements, excellent preservation of the remains, and the presence of spectacular partially or fully articulated skeletons (Fig. 1D and Fig. S2). The geological characteristics of the localities revealed that the assemblages were deposited in cavities formed as a result of a pseudokarstic process called piping [17] (Fig. 1B and Fig. S3). Fossiliferous accumulations associated with this kind of landform are practically unknown, so these fossil sites are exceptional both from paleontological and geological viewpoints.

Based on the geological evidence and the background information gained during the field seasons, we aim to determine a feasible scenario for the concentration of this uncommon assemblage from a number of plausible hypotheses (e.g., hydraulic transport of isolated remains or carcasses, denning activity, accidental fall in a pit, active entrance and entrapment in a pit). Works on particular taphonomic aspects of BAT-1 have been carried out previously ([21]–[25], Text S1), but a comprehensive and integrative study leading to a solid proposal of the mechanism of bone concentration was lacking. Taphonomic analyses on carnivore-rich fossil sites constitute a unique opportunity not only to understand the reasons behind the concentration of the individuals represented in the assemblages but also to comprehend different aspects of the ecology and behavior of these rare taxa.

### Geological Setting

Cerro de los Batallones is a structural butte located in the Madrid Basin (Fig. 1 and Fig. S1). The sedimentary sequence of the butte consists of three units that were deposited in terrestrial environments during the Early Vallesian (Late Miocene). From bottom to top, these units consist of 5 m-thick green to reddish magnesian swelling clays (Unit I), up to 9 m-thick deposits formed by white to grey lutites (sepiolite-palygorskite) with metric-sized chert nodules (Unit II) and up-to 5 m-thick heterolithic deposits constituted by an alternation of siliciclastic, marly and partially silicified carbonate beds [6], [17] (Fig. 1B and Fig. S1B). The particularity of the bone-bearing deposits, characterized by facies very similar to those observed in Unit III, is that they discordantly cut across the three units of the butte (Fig. 1B). Pozo et al. [17] proposed that Cerro de los Batallones fossil assemblages were deposited in cavities formed as a consequence of piping processes after the sedimentation of the aforementioned sedimentary sequence. This process involved the subsurface hydraulic erosion of clayey materials favored by concentrated infiltration along vertical discontinuities in the upper silicified sediments (Unit III) and the larger underground chert nodules embedded in Unit II. Cavities generated by piping processes normally have short survival-times, so they frequently collapse preventing their preservation in the geological record. On the contrary, Cerro de los Batallones cavities had a longer survival time due to the resistant nature of the silicified materials and chert nodules in which the cavities were partially carved. These resistant materials acted as the walls and roofs of the cavities, allowing their preservation and infilling, along with the accumulation of vertebrate remains. The cavities reached decametric diameters, with a pseudocircular shape in plan view and a top entrance (Fig. 1B). The sediments that filled the cavities consist of greenish marls and fine to coarse siliciclastics. Autochthonous carbonate deposits within the cavities, containing fossils of freshwater ostracodes, gastropods, charophytes, diatoms and sponges, imply that temporary fresh water shallow ponds developed at the cavity bottoms. Based on the complex geometry of the deposits, Pozo et al. [17] proposed that the cavities filled as a consequence of successive inputs of clastic materials, probably due to episodic sheetwash floodings. The ultimate filling of the cavities gave rise to the generation of discrete shallow ponds (“swamp zones”) at their top entries (Fig. 1B).

As the cavities filled, two different types of taphocoenoses were generated (Fig. 1B). The features of these two assemblages are: (1) lower level assemblages (LLA) are characterized by being embedded in the lower-middle part of the stratigraphic sequence of the butte (Unit II) and have a fossiliferous content overwhelmingly dominated by carnivorans remains; and (2) upper level assemblages (ULA) are embedded in the uppermost part of the butte sequence (Unit III) and possess a fossiliferous content that mainly consists of herbivore remains [6], [23]. The sedimentary matrix composition of BAT-1 ULA was similar to that of the LLA. Most of the BAT-1 ULA remains were destroyed during the mining activities, although a reduced number of fossils remained *in situ*. Bones from BAT-1 ULA yielded similar Rare Earth Element ( = REE) patterns to those analyzed in the LLA, which supports continuity in the burial environment and can be considered evidence of continuous filling of the cavities from the lower level to the upper level [23].

The geophysical prospection and direct observation allowed us to infer that BAT-1 LLA was deposited at a depth of 5 to 8 meters below the butte surface (where the opening to the cavity is inferred to have been). The pipe-like conduit that connected this assemblage to the ULA was made up of a sedimentary matrix with the same composition as the ULA and LLA, but was practically sterile in fossils. This conduit had a depth of ∼3 meters [6].

In 2002, a geophysical survey was carried out in the northeast wall (N140°E) of BAT-1 to further investigate the topography of the chamber and the internal geometry of its sedimentary filling (Fig. S3 and Text S1). It revealed the presence of a colluvial breccia at the bottom of the cavity, with embedded large boulders (0.5–0.7 m in diameter) of sepiolite, limestone and opal that came from the sedimentary sequence in which the cavity was carved and corresponded to fallen blocks from the wall or ceiling of the cavity. This colluvial wedge had a larger flank leaning towards the west and northwest.

## Materials and Methods

BAT-1 remains were dug up following standard techniques commonly used in vertebrate fossil excavations ([26], Text S1). The enormous amount of remains collected throughout the two excavation periods (1991–1993 and 2001–2008) made it unfeasible to carry out taphonomic observations on all of the elements. A total of 6,739 bones were selected for our taphonomic study; this subsample corresponds to all of the large mammal (Carnivora, Perissodactyla, Artiodactyla) material recovered in the first excavation period (1991–1993; this is ∼35% of the total large mammal remains found throughout all the field seasons). Only 142 fossils belong to BAT-1 ULA, because this level was almost completely destroyed during the sepiolite extraction. All necessary permits were obtained for the described field studies from the ‘Dirección General de Patrimonio Histórico de la Comunidad Autónoma de Madrid’. Cerro de los Batallones fossils are housed in the collections of the Museo Nacional de Ciencias Naturales-CSIC (Madrid, Spain).

Number of Identified Specimens (NISP), Minimum Number of Elements (MNE) and Minimum Number of Individuals (MNI) were calculated following Lyman [27]. Skeletal completeness of the individuals represented in the fossil site and Relative Abundance of particular skeletal elements per taxa were estimated with the formula by Andrews [28]:
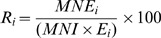
where R_i_ is the relative abundance of element i, MNE_i_ is the minimum number of element i in the sample, MNI is the minimum number of individuals in the taxon of interest, and E_i_ is the number of times that element i appears in a complete skeleton. To gauge whether or not there were bias in the skeletal representation of the different taxa (i.e., whether carcasses were deposited complete or not), we performed Kolmogorov-Smirnov tests comparing the observed skeletal representation to the expected skeletal representation (for the MNI estimated for each taxon). Significance for this test was established at the p = 0.01 level.

The susceptibility of the specimens to transport was investigated by the quantification of elements belonging to the transport groups defined by Voorhies [29] and complemented by Behrensmeyer [30]. Rose diagrams and stereographic projections were drawn to determine the possible existence of preferentially oriented remains. These diagrams were only drawn for BAT-1 LLA, because in BAT-1 ULA trend and plunge data were too scarce as to depict meaningful diagrams. These diagrams, along with Rayleigh’s tests (performed with the statistical software PAST version 2.17 [31]), served to determine whether bones showed a preferred or a random orientation.

The percentage of articulated, associated and isolated remains was also gauged, because it can provide clues regarding the relative amount of time elapsed between deposition and final burial.

Bone modification data were recorded after examining the taphonomic subsample under a binocular microscope. Bone alteration data (weathering, abrasion, trampling, carnivoran and rodent activity, root action and fragmentation) were analyzed following standard procedures and scales defined by Lyman [27], Behrensmeyer [32], and Alcalá [33]. Weathering measures the deterioration of bone due to physical and chemical agents and can be considered a proxy for the time elapsed between the death of the animal and final burial [32]. Abrasion refers to the erosion caused to the bones by the impact of sedimentary particles. It results in the smoothing and polishing of bones and is a proxy for the amount of transport that the bones underwent. As for the analysis of fragmentation, perpendicular and smooth fractures are typically the consequence of diagenetic processes, i.e., processes occurring after the burial of the remains such as sediment pressure or tectonic movements [27]. This type of fracture usually takes place when bone is dry and recrystallized or permineralized. In contrast, spiral and irregular or sawtoothed fractures are typical in relatively fresh, collagen-rich elements [27]. Different agents can produce fresh fractures such as carnivoran activity, trampling or transport.

Bone modification data from BAT-1 LLA and ULA were compared using chi-square tests or the analogous Fisher’s exact test (in those cases when n<5). Significance was established at the p = 0.01 level.

## Results

The main taphonomic characteristics of Batallones-1 LLA and ULA are summarized in Table 1.

**Table 1:** Main taphonomic features of the large mammal remains from BAT-1 LLA and ULA.

	BAT-1 LLA	BAT-1 ULA	χ2/Fisher’s exact test
**Assemblage** **data**			
NISP	6597	142	
MNI	81	10	
Number of large mammal species	19(a)	7	
Predominant taxonomic group(b)	Carnivores (98.32%)	Herbivores (80.55%)	
Body size (kg)	10^0^–10^3^	10^1^–10^3^	
Age structure	82.98% adult(c)	ndet	
Skeletal articulation	9.42% art.	0% art.	
Common Voorhies groups	I-II, II and III	III(d)	
**Spatial data**			
Assemblage area	∼87 m^2^	∼10 m^2(e)^	
Fossil density	205.3 specimens/m^2^	8 specimens/m^2^	
Bone orientation	Preferred	ndet	
**Bone modification data**			
Weathering	0.11%	2.36%	s
Abrasion	0.11%	1.57%	ns
Carnivore marks(f)	0.53%	0%	ns
Root marks	0.06%	17.97%	s
Trampling marks	0%	0%	-
Rodent marks	0%	0%	-
Breakage			
Complete and almost complete bones (all bones)	78.62%	44.27%	s
Complete and almost complete bones (only long bones)	80.57%	22.92%	s
Fracture angle (only long bones)	Mainly perpendicular	No clear pattern	s
Fracture surface (only long bones)	Mainly smooth	No clear pattern	ns
Sediment compaction	19.27%	4.84%	s

NISP = Number of Identified Specimens, MNI = Minimum Number of Individuals, ndet = non-determined.

(a) This is the total number of large mammal species from BAT-1 LLA including a bovid and a cervid represented by very few remains and recovered in the 2001–2008 excavation period.

(b) Indetermined elements were not included in this quantification.

(c) Age structure of BAT-1 LLA is based on the four most common species (Fig. S4).

(d) Voorhies groups in BAT-1 ULA are only based on *Hipparion* sp. remains due to the scarcity of remains for the rest of taxa (Fig. S10).

(e) This is a minimum estimate because most of this assemblage was destroyed.

(f) We include bones that have suffered both physical damage and chemical (digestion) damage. Ns, = non significantly different, s = significantly different p<0.01.

### BAT-1 LLA Assemblage Data

The total number of large mammal species of BAT-1 LLA is 19 (Table 1). The NISP of the taphonomically analyzed sample (1991 to 1993 excavations) is 6,597 (Table 2). Carnivoran remains dominate the assemblage with a NISP of 6,128 (92.89%; 98.32% if indetermined remains are not considered) and a MNI of 71 (Table 2). The most abundantly represented taxa are the two sabertoothed cats, *Promegantereon ogygia* and *Machairodus aphanistus*, with NISP of 2,231 and 1,600 and MNI of 21 and 18 respectively. Thus, their remains (and individuals) practically constitute half of the large mammal remains from BAT-1 LLA. Based on quantifications from the field notes, we have preliminarily inferred that the total MNI for these two species increases to 50 and 23 respectively, if the 2001 to 2008 collection is regarded. Herbivore material consists of remains of perissodactyls–represented by an equid and two rhinocerotids–and artiodactyls–represented by three musk deer species and a suid (Table 2). Herbivore NISP and MNI are 105 and 10 respectively, comprising only 1.59% of the NISP of the assemblage and 12.35% of the MNI of the assemblage.

**Table 2:** Number of Identified Specimens (NISP), Minimum Number of Elements (MNE) and Minimum Number of Individuals (MNI) of the large mammal taxa recovered in BAT-1 LLA.

	Family/Order	NISP	%	MNE	%	MNI	%
*Promegantereon ogygia*	Felidae/Carnivora	2231	33.82	2154	44.44	21	25.93
*Machairodus aphanistus*	Felidae/Carnivora	1600	24.25	1543	31.83	18	22.22
*Protictitherium crassum*	Hyaenidae/Carnivora	635	9.63	619	12.77	10	12.35
*Magericyon anceps*	Amphicyonidae/Carnivora	395	5.99	366	7.55	10	12.35
*Simocyon batalleri*	Ailuridae/Carnivora	101	1.53	100	2.06	3	3.70
Felinae indet.	Felidae/Carnivora	125	1.89			4	4.94
Mustelidae/Mephitidae indet.	Mustelidae-Mephitidae/Carnivora	127	1.93			5	6.17
Carnivora indet.	Carnivora	914	13.85				
*Hipparion* sp.	Equidae/Perissodactyla	10	0.15	10	0.21	2	2.47
*Aceratherium incisivum*	Rhinocerotidae/Perissodactyla	24	0.36	24	0.50	2	2.47
Rhinocerotidae indet.	Rhinocerotidae/Perissodactyla	29	0.44				
*Micromeryx soriae*	Moschidae/Artiodactyla	12	0.18	12	0.25	3	3.70
*Hispanomeryx* sp. cf. *H. duriensis*	Moschidae/Artiodactyla	3	0.05	3	0.06	1	1.23
*Micromeryx* sp. ‘large size’	Moschidae/Artiodactyla	4	0.06	4	0.08	1	1.23
Moschidae indet.	Moschidae/Artiodactyla	5	0.08				
*Microstonyx* sp.	Suidae/Artiodactyla	13	0.20	12	0.25	1	1.23
Ruminantia indet.	Artiodactyla	3	0.05				
Artiodactyla indet.	Artiodactyla	2	0.03				
Indetermined		364	5.52				
	**TOTAL CARNIVORA**	6128	92.89	>4782	98.66	71	87.65
	**TOTAL PERISSODACTYLA**	63	0.95	>34	0.70	4	4.94
	**TOTAL ARTIODACTYLA**	42	0.64	>31	0.64	6	7.41
	**TOTAL INDETERMINED**	364	5.52				
	**TOTAL**	6597		>4847		81	

MNE could only be estimated for well-defined taxa (species level or monospecific taxa). Indet. = indetermined.

Isolated bones dominate BAT-1 LLA (79.46%). Articulated bones make up 9.42% of the assemblage, but the presence of some partially or fully articulated skeletons is noteworthy (Table 1, Fig. 1D and Fig. S2).

**Figure 2:**
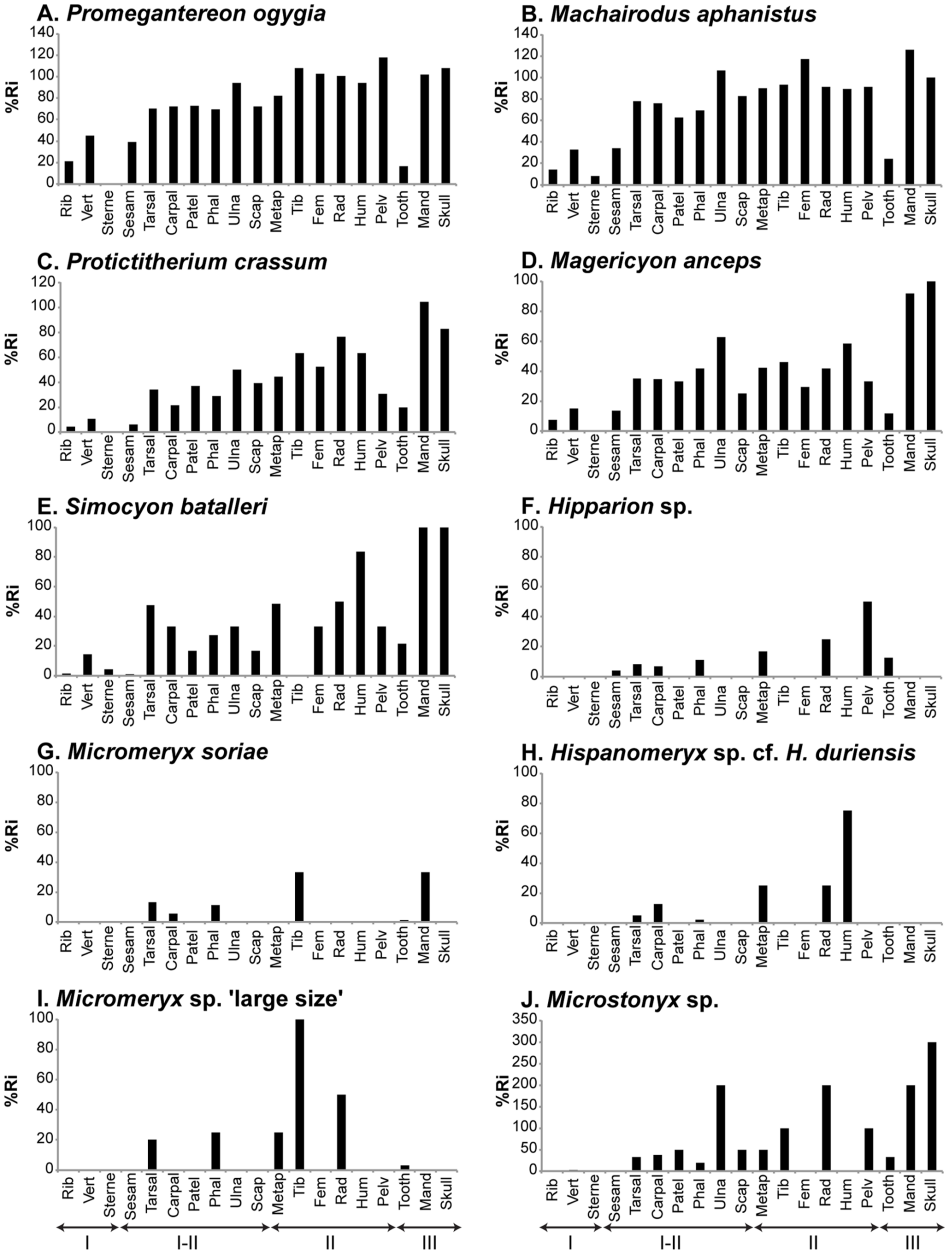
Skeletal element proportions expressed as Relative Abundance (%Ri) in BAT-1 LLA. Transport groups are given in roman numerals following Voorhies [29] and Behrensmeyer [30]. Group I: inmediately transported elements, Group II: elements transported gradually, Group III: lag deposit. Group I-II is an intermediate category. Note that the y-scale changes across the plots. Vert = vertebra, sterne = sternebra, sesam = sesamoid, patel = patella, phal = phalanx, scap = scapula, metap = metapodial, tib = tibia, fem = femur, rad = radius, hum = humerus, pelv = pelvis, mand = mandible.

Skeletal completeness was calculated for those taxa that in Table 2 have an estimation of their MNE, except for the rhinoceros *Aceratherium incisivum*. Two species of rhinoceroses have been recognized in BAT-1 LLA; however, they are not distinguished in the 2001–2008 field notes, making it impossible to obtain an accurate estimation of the remains belonging to each species. Figure 2 shows that the relative abundance of some elements goes beyond 100% even though 100% should be the maximum value; this is an artifact produced because some 2001–2008 elements are represented by several specimens due to breakage. For all of the analyzed taxa, only some elements are completely represented in the fossil assemblage (Fig. 2). Vertebrae, ribs, sesamoids (other than patella) and sternebrae are difficult to assign with confidence to a particular species because they lack resolutive characters. Consequently, it is possible that they can be mistakenly assigned to a particular species or left as an element without a taxonomic adscription (either Carnivora indet. or Indet.). And in fact, we see that while all these elements are not well represented for any taxon (Fig. 2), they are abundant in the estimates for Carnivora indet. or Indet. (Fig. S5 and Text S1).

However, it must be highlighted that the completeness percentage for the two sabertoothed cats is very high, and the Kolmogorov-Smirnov test indicates that there is no significant anatomical bias when we compared the remains recovered in the fossil site to those expected for complete skeletons (Table 3). Thus, we consider that complete *Promegantereon ogygia* and *Machairodus aphanistus* skeletons accumulated in the fossil site. At the significance level fixed in this work (p = 0.01), the same is true for the rest of the carnivorans; however, at a p = 0.05 significance level, the skeletons of these taxa would be underrepresented (but see Discussion below). Except for the suid *Microstonyx* sp., skeletal representations of the herbivore taxa exhibit statistically significant departures from the expected values, which suggests that complete skeletons were not deposited in BAT-1 LLA (Table 3).

**Table 3:** Skeletal completeness for BAT-1 LLA well-defined taxa.

		Kolmogorov-Smirnov test	
	% Skeletal completeness	D	p
*Promegantereon ogygia*	75.80	0.33	0.31
*Machairodus aphanistus*	78.40	0.4	0.14
*Protictitherium crassum*	35.99	0.53	0.02
*Magericyon anceps*	40.79	0.53	0.02
*Simocyon batalleri*	35.54	0.53	0.02
*Hipparion* sp.	10.69	0.67	*<0.01*
*Micromeryx soriae*	6.15	0.88	*<0.01*
*Hispanomeryx* sp. cf. *H. duriensis*	5.34	0.81	*<0.01*
*Micromeryx* sp. ‘large size’	13.59	0.63	*<0.01*
*Microstonyx* sp.	39.74	0.25	0.63

Teeth (only in the case of the carnivorans), vertebrae, ribs, sesamoids (other than patella) and sternebrae were removed for the estimation of ‘% Skeletal completeness’ (see Text S1). The Kolmogorov-Smirnov test compares the observed number of skeletal elements and the expected number of elements for the MNI per taxa represented in BAT-1 LLA. p>0.01 = no significantly different, p<0.01 = significantly different (in italics).

Voorhies groups I-II, II and III are the most represented for the carnivores in general (Fig. 2). For the herbivores, elements belonging to Voorhies groups I-II and II are the most abundant. Voorhies group III is well represented for *Microstonyx* sp. Thus, there is coexistence of the whole range of transport groups proposed by Voorhies [29] in this assemblage.

### BAT-1 LLA Spatial Data

**Figure 3:**
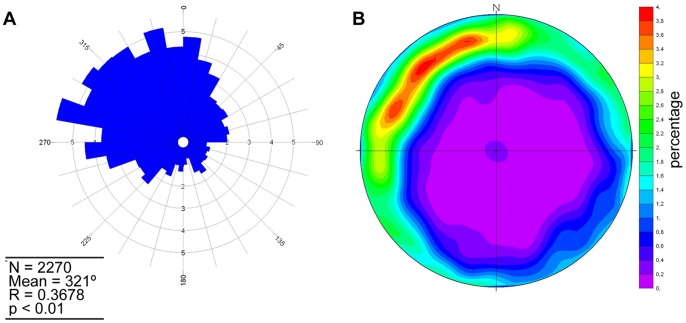
Spatial distribution of the fossil material from BAT-1 LLA. A, Rose diagram. Note that Rayleigh’s test points to a preferred orientation of the fossils (i.e., p<0.01); B, Contour diagram of data presented in a stereographic projection, the scale is indicative of the percentage of fossils showing a particular plunge and trend. The analyses are based on fossils showing a longer axis.

Skeletal elements exhibited a preferred orientation: most of the BAT-1 LLA specimens yielded a plunge direction between 270° and 0°, a moderate number of bones had plunge directions that go from 0° to 90° or from 180° to 270°, and a low number of remains tilted towards the portion that ranges from 90° to 180° (Fig. 3). The analysis of the data in separate grids revealed that the orientation varied slightly in a fan-like pattern (Fig. S6). The analysis of distribution of bones in a vertical section oriented SE-NW shows that they laid over a surface that sloped to the northwest (Fig. S7).

### BAT-1 LLA Bone Modification Data

Bones showing no weathering alteration (Stage 0) predominate in BAT-1 LLA (99.89%; Table 1 and Fig. S8). None of the remains display weathering stages 4 or 5. Most of the sample is classified in abrasion stage 0 (99.89%), which means that rounding and polishing of bones due to abrasion is practically absent. Carnivore marks occur in only 20 specimens (0.30% of the studied sample). The most common carnivore marks are pits and scores. 15 specimens (0.23%) show evidence of having been digested. Of these, 11 remains belong to Moschidae species [24]. Four bones (0.06%) exhibit root marks. Trampling and rodent marks are absent in BAT-1 LLA (Table 1).

BAT-1 LLA remains show high integrity. Complete and almost complete bones (only missing some bone chips) make up 78.62% of the analyzed sample (Table 1 and Fig. S9). 80.57% of the long bone remains are complete or almost complete. Broken long bones mainly display perpendicular and smooth fractures and maintain the fragments adjacent to one another (Table 1 and Fig. S9).

**Figure 4:**
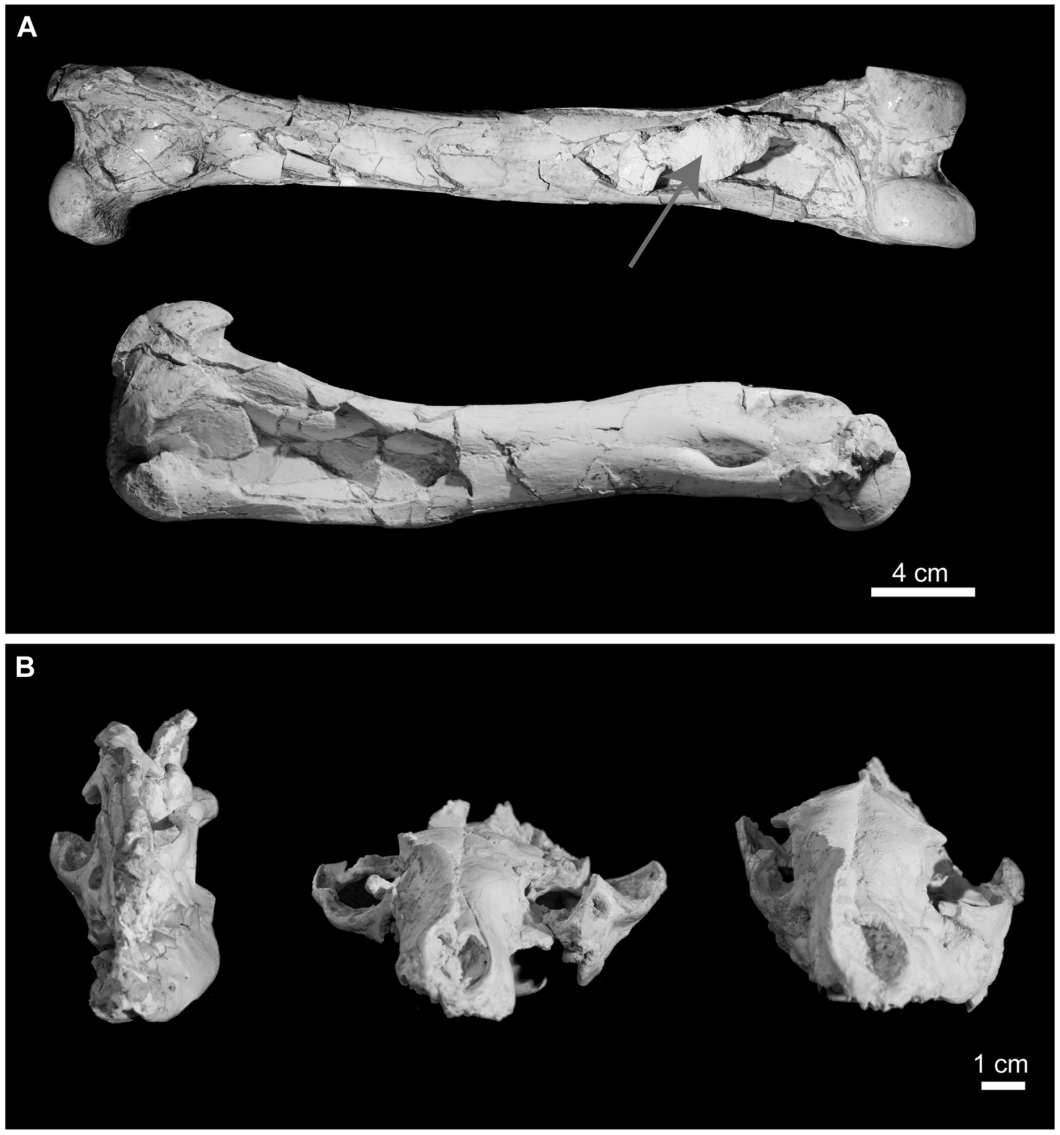
Bone modifications due to sediment compaction in BAT-1 LLA. A, Bone collapse in femur B-7056 (top) and humerus B-2721 (bottom) of *Machairodus aphanistus.* Note that a fragment of another bone is encrusted in the distal part of the femur diaphysis (grey arrow). Collapse in that area of the femur was probably caused by the bone-bone contact; B, Deformation of *Protictitherium crassum* skulls. Left: skull B-2804; center: skull B-2802; right: skull B-2889.

19.27% of the remains exhibit modifications due to sediment compaction, such as encrustation of sedimentary particles or other bones on them, plastic deformations and bone collapse. These modifications mainly occur in bones with thinner walls or hollow or flat morphologies like the skull, the proximal portion of the humerus or the pelvis (Table 1 and Fig. 4).

### BAT-1 ULA Taphonomic Results

Seven large mammal species have been recognized in BAT-1 ULA (Table 1 and Table 4). Herbivore remains are more abundant than carnivore remains and make up 61.27% of the total NISP (80.55% if indetermined remains are not regarded). The equid *Hipparion* sp. is the most abundant taxon, represented by a NISP of 76 and a MNI of 4 (Table 4). The scarcity of remains in this assemblage prevented the performance of some analyses, such as age profile or spatial analyses. None of the BAT-1 ULA specimens were found articulated or associated (Table 1). None of the taxa are represented by the number of remains that would be expected in complete skeletons (Table S1 and Fig. S10).

**Table 4:** Number of Identified Specimens (NISP), Minimum Number of Elements (MNE) and Minimum Number of Individuals (MNI) of the large mammal taxa recovered in BAT-1 ULA.

		NISP	%	MNE	%	MNI	%
*Magericyon anceps*	Amphicyonidae/Carnivora	7	4.93	7	7.61	1	10
*Protictitherium crassum*	Hyaenidae/Carnivora	2	1.41	2	2.17	1	10
*Simocyon batalleri*	Ailuridae/Carnivora	2	1.41	2	2.17	1	10
Carnivora indet.	Carnivora	10	7.04				
*Hipparion* sp.	Equidae/Perissodactyla	76	53.52	70	76.09	4	40
Rhinocerotidae indet.	Rhinocerotidae/Perissodactyla	1	0.70	1	1.09	1	10
*Microstonyx* sp.	Suidae/Artiodactyla	2	1.41	2	2.17	1	10
*Tetralophodon longirostris*	Gomphotheriidae/Proboscidea	8	5.63	8	8.70	1	10
Indetermined		34	23.94				
	**TOTAL CARNIVORA**	21	14.79	>11	11.96	3	30
	**TOTAL PERISSODACTYLA**	77	54.23	71	77.17	5	50
	**TOTAL ARTIODACTYLA**	2	1.41	2	2.17	1	10
	**TOTAL PROBOSCIDEA**	8	5.63	8	8.70	1	10
	**TOTAL INDETERMINED**	34	23.94				
	**TOTAL**	142		>92		10	

MNE could only be estimated for well-defined taxa (species level or monospecific taxa). Indet. = indetermined.

Weathering alteration is scarce (97.64% of the remains show weathering stage 0), although significantly higher than that detected in the LLA (Table 1). Only 1.57% of the remains have alteration due to abrasion. None of the specimens exhibit trampling, carnivore or rodent marks. Twenty three bones (17.97% of the examined sample) show root marks, an estimate significantly different from the LLA values.

Breakage is more common in bones from the ULA than from the LLA. Complete and almost complete bones represent 44.27% of the total assemblage (Table 1 and Fig. S11). Elements that exhibit modifications due to compaction in the LLA, such as the radius, the third phalanx, the ulna, the tibia or the pelvis, are not affected by this modification in the ULA and, in general, this is a less important taphonomic agent than observed in the LLA.

## Discussion

BAT-1 LLA was deposited at the bottom part of a cavity that had a single upper outlet, as evidenced by the geological and geophysical studies and as observed throughout the course of the excavations (Fig. 1B and Fig. S3). This geological context restricts the number of possible scenarios, and all open-air settings are discarded from the formation hypotheses regarded in this study. Different processes can lead to the accumulation of remains in caves. The next hypotheses for the concentration of BAT-1 LLA remains are evaluated: transport of skeletal elements or carcasses into the cave, denning activity of carnivores, accidental fall, and intentional entrance. Table 5 reports a number of predictions that should be satisfied had BAT-1 LLA originated as the consequence of any of these hypotheses.

**Table 5:** Expected features of fossil assemblages accumulated under different scenarios.

	Hydraulic transport				Den		Natural trap			
	Isolated skeletal elements		Entire carcasses				Accidental		Intentional	
Carnivore abundance	Low	N	Low	N	Moderate	N	Low-Moderate	N	High	Y
Herbivore abundance	High	N	High	N	Moderate-High	N	High	N	Low	Y
Mortality profile	U or L-shaped	N	U orL-shaped	N	U-shaped	N	L-shaped	N	Depends onbehavior	Y
Completeness of skeletons	Incomplete	Partial	Complete	Partial	Complete/Incomplete	Y	Complete	Partial	Complete	Partial
Associated and articulated elements	Absent	N	Present	Y	Present	Y	Present	Y	Present	Y
Hydraulic transport evidence(a)	High	N	Variable		Low	Y	Low	Y	Low	Y
Bone orientation	Preferred	Y	Variable		Variable		Variable		Variable	
Carnivore activity(b)	Variable		Variable		High	N	Variable		Variable	
Weathering alteration	Variable		Low	Y	Low	Y	Low	Y	Low	Y
Trampling marks	Variable		Low	Y	High	N	Variable		Variable	
Root alteration	Variable		Low	Y	Low	N	Low	Y	Low	Y
REE pattern	Heterogeneous	N	Variable		Homogeneous	Y	Homogeneous	Y	Homogeneous	Y

We have indicated whether BAT-1 LLA meets (Y), does not meet (N) or partially meets (‘Partial’) the predictions for each scenario. ‘Variable’ means that the taphonomic agent is not intrinsic to the scenario but depends on the particular conditions under which each assemblage was accumulated and, therefore, can vary.

(a) Evidenced by the presence of particular Voorhies groups, the degree of fragmentation of the remains and the abrasion and polishing displayed by the elements.

(b) Evidenced by carnivore tooth marks, digested remains, spiral breakage of bone, predominance of meaty body portions and presence of coprolites.

REE = Rare Earth Elements.References: [5], [26], [27], [29], [34]–[39].

To start with, plausible scenarios to explain the formation of the assemblage must necessarily take into account processes leading to such a selective concentration, i.e., a concentration massively dominated by carnivoran specimens and individuals. Besides, the mortality profiles exhibited by the four most common taxa (the two sabertoothed cats, the hyaenid and the amphicyonid) do not correspond to the classic attritional (U-shaped) or catastrophic (L-shaped) death modes and reduce the number of possible hypotheses as well. The two sabertoothed cats and the hyaenid have mortality profiles dominated by prime adults. On the other hand, juveniles prevail in the amphicyonid age profile ([25]; Fig. S4). Other lines of evidence reject any type of mass mortality accumulation. Domingo et al. [23] carried out REE analyses of fossil bones from Batallones-1. The negative Ce anomaly determined for all the analyzed samples implied that the water bodies or ponds present at the chamber during the deposition of BAT-1 LLA were oxic. The oxic nature of the ponds suggests that a high number of decaying carcasses did not occur at the same time, so the assemblage is not the consequence of one or several mass mortality events. Rather, incorporation and death of animals must be considered as a process that occurred over a relatively protracted period of time [23].

Under conventional conditions, hydraulic transport of isolated remains or carcasses from the outside to the interior of the cavity would result in an assemblage dominated by herbivores remains (Table 5). Depending upon the circumstances that led to the death of the individuals, either a U-shaped or L-shaped profile would be expected for a transported assemblage. A profile dominated by prime adults, as seen for some taxa here, is highly unlikely in scenarios that have transport as their main concentration driver. Apart from this, other predictions are not met under the transport hypothesis (Table 5). Had elements arrived in the cavity disarticulated and as a consequence of hydraulic transport, the number of broken and abraded bones should be higher and, because elements could come from different locations, a heterogeneous REE pattern would be expected. Most of the bones from BAT-1 LLA exhibit similar REE patterns, which implies that there was no mixing of bones with different provenances, i.e., bones are not reworked [23]. A transported association would also show a predominance of skeletal elements more prone to be transported (Voorhies Groups I and I-II); nevertheless, Group III elements are very abundant in BAT-1 LLA. Thus, we exclude hydraulic transport as the agent for the accumulation of carcasses and remains in BAT-1 LLA.

Although the carnivoran MNI can be considered as moderately high in assemblages originated by denning activity (from 6% to 53%; [35], [36], [39]; Table 5), the percentages are far from the estimates obtained in BAT-1 LLA. In general, prey species dominate the bone concentration in dens. The carnivoran assemblage recovered at Friesenhahn Cave [37], a bone accumulation produced by the denning activity of the sabertoothed cat *Homotherium serum*,was dominated by juvenile and old individuals of this cat (U-shaped profile). This mortality pattern differs from those obtained for the two sabertoothed cats and the hyaena from BAT-1 LLA. Dens have other distinctive features not observed in the LLA, such as abundance of tooth-marked and spirally-broken bones and an outlet that provided easy ingress and egress. For all these reasons, the concentration of remains as a consequence of denning activities is ruled out.

**Figure 5:**
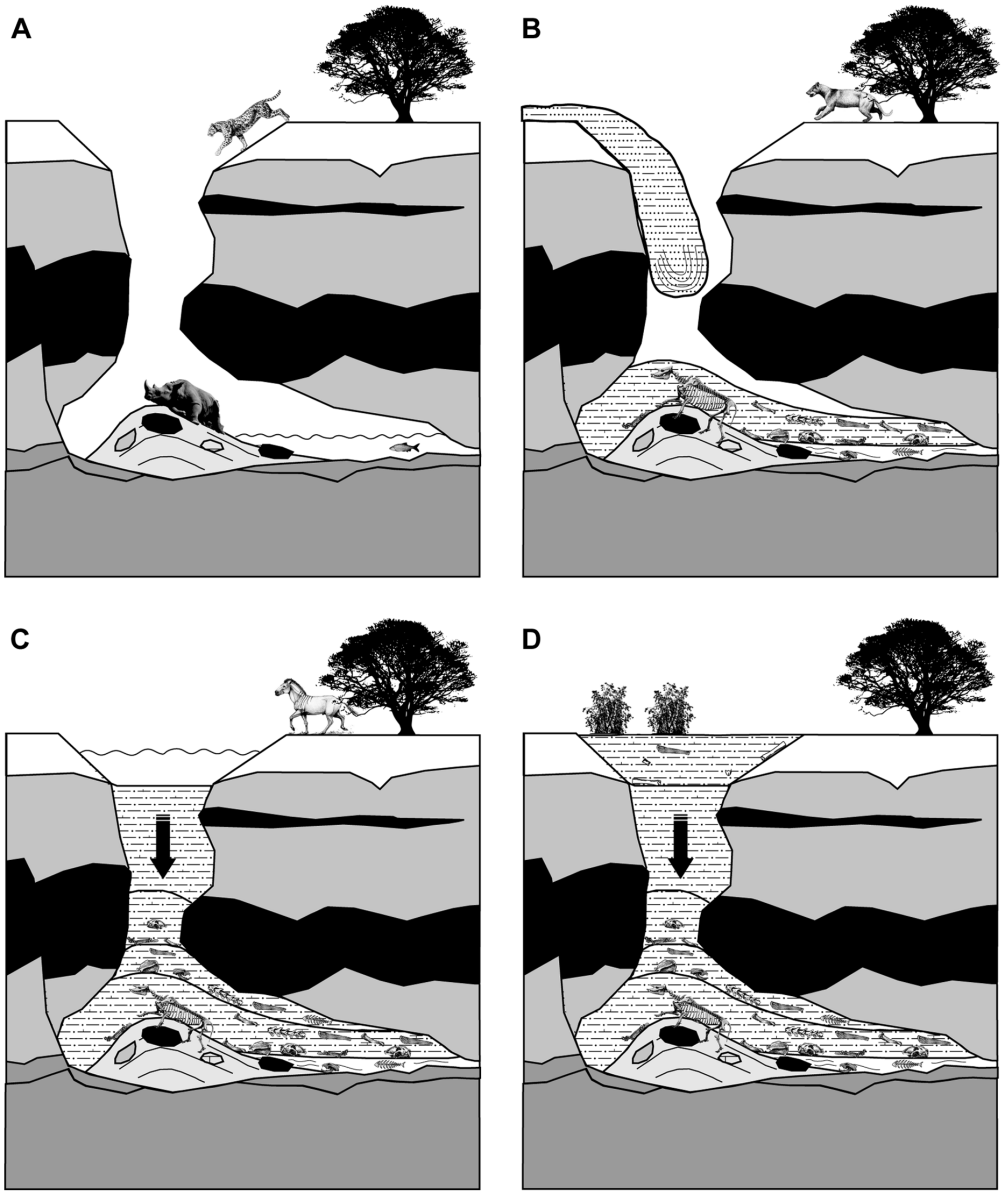
Sequence of events in the formation of BAT-1 LLA and ULA. A, Carnivorans intentionally entered into the cave attracted by trapped/death animals or water; B, Episodic floods filled the cavity and buried remains in different articulation stages; remain orientation was the result of their adaptation to a colluvial breccia; elements in the chamber are protected from different taphonomic alterations; repeated carnivoran entrances through time; C, Lithostatic pressure produced breakage, collapse and deformation of remains; final stages of the filling of the chamber: the location is not a carnivore trap anymore; D, Deposition of elements from the ULA in an uncertain taphonomic context (entrapment of herbivores?).

There are two modes of faunal concentration in natural traps developed in caves: accidental falls (passive accumulation) and intentional entrance (active accumulation). In principle, pitfall concentrations are fundamentally unselective, i.e., a diverse unbiased fauna with all age classes (L-shaped pattern) is expected to be represented in the accumulation [e.g. 34,36; Table 5]. Carnivoran remains are far less abundant in pitfalls than what is observed in BAT-1 LLA; thus, we suggest that the intentional (active) entrances of carnivores, attracted by animal carcasses (or water), was the main mechanism of accumulation (Table 5 and Fig. 5). Weakened or dead carnivore individuals would become additional bait for the trap. The low number of herbivores suggests that the opening to the trap was well visible and avoided; therefore, accidental falls were not common. However, we do not discount that some accidental trapping happened. In fact, this could be the fate of an individual of the rhinoceros species *Aceratherium incisivum* which is represented by a complete skeleton (Fig. S2C; see further explanation for the deposition of herbivore remains in Text S1).

Age profiles are additional evidence that trapping of carnivorans was mainly active and not accidental. An interesting aspect reflected in the age classes distribution of the four most common taxa is that entrance was controlled by behavioral factors. The prime-adult dominated mortality pattern shown by *Promegantereon ogygia*, *Machairodus aphanistus* and *Protictitherium crassum* would respond to a deliberate entrance into the cavity, to get ‘easy’ food and water, of individuals that were struggling due to their inexperience in getting resources [11], [25], [40]. In modern ecosystems, mortality among prime adult individuals is not rare and is related to their proneness to search for resources in dangerous locations mainly during the stressful period of dispersal ([25] and references therein). The abundance of *Magericyon anceps* juvenile individuals could be due to the existence of a close bond between cubs and adults (presumably females) in the same fashion as ursids today [40]. Although we have discarded a den as explanation for the origin of the assemblage, it is still likely that the amphicyonids were looking for a place to den but, instead, became trapped.

Domingo et al. [25] analyzed the presence of osteopathologies and broken canines in life for the four most abundant taxa from Batallones-1 LLA in order to determine whether individuals with poor skeletal physical condition were preferentially attracted and trapped. Occurrence of bone pathologies and broken canines in life were compared to modern healthy carnivoran populations, and results did not differ significantly from the modern populations patterns. Therefore, there was not preferential entrapment of individuals having poor skeletal health [25].

It is uncertain whether the carnivorans died as a consequence of the fall *per se* or remained alive for some time, eventually dying after progressive weakening. Low incidence of carnivore alteration and absence of trampling marks seem to point towards an instantaneous death in the fall. Nevertheless, we consider that it is difficult to reconcile the facts that carnivorans were intentionally entering the cavity but dying in the jump. Carnivores could be struggling for getting resources but it is unlikely that they risked their lifes in the jump. Most probably, carnivores got trapped and remained alive for some time. Low incidence of carnivore marks could be the result of low exploitation of carcasses because there was plenty of meat or because carnivorans died quickly after entrapment. Also, it is possible that carnivores were searching for water during drought periods and not necessarily for food. Further research is needed in order to ascertain the causes of death of carnivorans inside the cavity but exhaustion, hypothermia or poisoning from drinking water or toxic gases are options to consider.

Most of the predictions for the intentional entrance hypothesis are met by the taphonomic features of this assemblage (Table 5). The Kolmogorov-Smirnov test performed to assess the completeness of the skeletons points towards the deposition of complete carcasses at least for the most abundant taxa (i.e., the two sabertoothed cats, the amphicyonid, the hyaenid and the ailurid) (Table 3). *Magericyon anceps*, *Protictitherium crassum* and *Simocyon batalleri* are missing more bones than the two sabertoothed cats (Table 3). Nevertheless, *Magericyon anceps*, *Protictitherium crassum* and *Simocyon batalleri* remains do not exhibit evidence of higher levels of transport, breakage or carnivore damage (i.e., NISPs showing carnivore modification are very low for these three carnivorans as well as for the two sabertoothed cats, ranging from 0% to 0.47%) than the sabertoothed remains that could point to a different accumulation mode for the elements of these taxa. Besides, REE patterns are similar for all the remains belonging to these taxa [23]. For all these reasons, and because some partially or fully articulated skeletons of the amphicyonid, the hyaenid and the ailurid have been found in BAT-1 LLA (Fig. 1D and Fig. S2B), we believe that the concentration mode for these three taxa was the same as for the two sabertoothed cats; i.e., active entrance of carnivorans in the cavity and inability to make their way out of it. The amphicyonid is represented by many juvenile individuals, so it is possible that some bone loss could occur due to the destruction of low density young bones. Also, differential destruction could be the reason behind the lower skeletal completeness of *Protictitherium crassum* due to its small body size and, therefore bone size (*Protictitherium crassum* was the size of an African civet [40]). *Simocyon batalleri* is approximately the same size as *Promegantereon ogygia* so, although we consider that complete carcasses were deposited in the cavity, it is uncertain why this taxon has lower degree of skeletal completeness than the sabertoothed cat.

As the individuals died, their carcases acquired a spatial distribution conditioned by the topography and basal colluvial accumulations of the cavity. The preferred orientation exhibited by BAT-1 LLA remains is consistent with their deposition in a sedimentary matrix that capped the colluvial wedge (with the northwest flank more developed) (Fig. 5).

In the context of a cavity, where humidity and temperature conditions are stable and sun light is restricted, bones can remain unburied for a long time without undergoing heavy weathering [36]. In BAT-1 LLA, most of the bones were disarticulated, which implies that immediate burial of carcasses did not frequently occur but enough time elapsed for the carcasses to became disarticulated. As mentioned above, the filling of the cavity happened episodically when repeated floods brought clastic material into the chamber. Totally or partially articulated skeletons of recently died animals were buried as articulated elements, together with isolated and associated remains of individuals that died longer before (Fig. 5); this mixture of elements in different disarticulation stages constitutes further evidence that entrance of individuals happened over a protracted period of time. In general, bones from BAT-1 LLA show a very good preservation state, with compaction of bones due to sediment loading representing the taphonomic process that affected the largest number of remains (Table 1 and Fig. 4).

Most of BAT-1 ULA was destroyed during the sepiolite quarrying. This translates into a poorer knowledge of the taphonomy of this assemblage and, therefore, of the concentration mode. Geological and geochemical evidence (i.e., similar REE patterns for bones from BAT-1 ULA and LLA) suggests that the cavity filled in a continuous manner and this assemblage corresponded to the final stages of the filling of the cavity [23]. Its taxonomic composition, with a larger abundance of herbivores, implies that at this stage there was not a carnivore trap anymore (Fig. 5). Probably, this assemblage is more related to open-air accumulations (with the presence of swampy zones) and, in fact, some of its taphonomic attributes point in this direction (e.g., higher abundance of weathered and root-affected remains, lower incidence of sediment compaction; Table 1).

The occurrence of completely or partially articulated skeletons of numerous herbivore taxa in better preserved ULAs of the butte (e.g., BAT-2 and BAT-10 fossil sites; [6]) leads us to suspect that an entrapment process cannot be discarded in this level. In this case, the entrapment would have mainly affected large herbivore taxa (proboscidean, rhinoceroses, sivatherine giraffes and hipparionine horses). Future taphonomic analyses in ULAs concentrations will shed light on the taphonomic history undergone by mammalian remains in these assemblages.

Although often neglected, detailed taphonomic studies are of great utility to clarify the formation scenario of fossil sites. The importance of fulfilling comprehensive taphonomic analyses is accentuated when exceptional fossil sites are discovered. Here, we have described the taphonomic history of one of the very few examples of carnivoran-dominated assemblages known in the fossil record of mammals. Because taphonomy is closely related to paleoecology, our analyses have allowed us to enhance the knowledge on aspects of the ecology and behavior of taxa (mainly carnivorans) that are uncommon in the fossil record..

### Conclusions

Batallones-1 locality (Madrid Basin, Spain) combines two features that are unusual in the fossil record of mammals. First, its lower level assemblage is dominated by carnivoran remains (the expected herbivore to carnivore ratio is reversed with more than 90% of the skeletal elements belonging to diverse carnivoran taxa), and therefore offers unique opportunities to gain insight into the diversity and ecology of past carnivoran guilds. Second, the accumulation of remains took place in a piping-generated cavity, a geomorphological landform rarely associated with the development of fossil sites.

In Batallones-1 LLA, the most abundant taxa are two species of sabertoothed cats. The mortality profiles are the result of the behavioral inclinations of the analyzed taxa. We formulate a scenario that takes most of the taphonomic evidence into account: the active entrance of carnivorans into the cave, attracted by food or water, and their subsequent entrapment was the main driver for the bone accumulation in BAT-1 LLA. This entrapment happened over a prolonged period of time, as implied by the absence of mass mortality profiles and high amount of isolated remains. The scarcity of herbivore individuals implies that the opening to the cave was well visible and avoided.

In the protective environment of the cavity, skeletal elements retained their integrity and good preservation. The most extensive taphonomic alterations on fossils from BAT-1 LLA are the result of the sediment compaction.

In turn, BAT-1 ULA shows a predominance of herbivore remains. We suggest that the deposition of remains in the ULA happened during the final stages of the cavity filling. The two levels of the fossil site of BAT-1 must be regarded as consecutive faunal concentrations, their differences in taxonomic composition being the consequence of the evolution of the site from a trap towards a more open surface-like deposit. BAT-1 ULA was largely disturbed by the quarrying works, so taphonomic analyses on other ULAs from Cerro de los Batallones are being pursued in order to discern the factors that produced the accumulation of large herbivores in these assemblages.

## Supporting Information

Figure S1 **Cerro de los Batallones and Batallones-1 geologic and taphonomic context.** A, Location of Cerro de los Batallones within the Madrid Basin (Modified from Calvo et al. [18]); B, Stratigraphic column of Cerro de los Batallones (Modified from Morales et al. [6]). Location of the fossil bones is not indicated since they are embedded in a sedimentary unit that discordantly cuts the three main units of the butte; C, Plant view of the grid system used in the excavation of Batallones-1. Each of the grids is named after a letter and a number (indicated on the border of the drawing) and has 2×2 m dimensions. Numbers within each of the grids is the total amount of large mammal fossils recovered throughout all the field seasons (1991–1993 and 2001–2008). The star corresponds to the (0, 0) coordinate for the X and Y of the system used to locate the fossils. Question marks indicate that the limits of the Batallones-1 LLA have not been found in that area of the fossil site.
(PDF)

Figure S2 **Fossils from BAT-1 LLA.** A, Skull and mandible of the sabertoothed cat *Machairodus aphanistus* (B-5445); B, Articulated foot of the amphicyonid *Magericyon anceps*. The scale represents 5 cm; C, Articulated skeleton of the rhinoceros *Aceratherium incisivum*. Note that the skull is disarticulated and displaced.
(PDF)

Figure S3 **Geophysical profile of BAT-1 LLA.** A, Electrical resistivity tomography 2D inverse model performed parallel to the northeast wall of BAT-1 LLA (vertical section) (Modified from Morales et al. [6]). The fossiliferous content of the fossil site was prospected in the 1991–1993 period in the location marked as ‘exploratory trench’. The arrangement of the electrodes is given in the horizontal axis. The electrodes were 1 meter away from each other. The depth of the survey (in m) is provided in the vertical section; B, Geological interpretation of the geophysical model.
(PDF)

Figure S4 **Age structure of the four most common taxa from Batallones-1 LLA.** Age structure of the taxa analyzed (*Promegantereon ogygia*, *Machairodus aphanistus*, *Protictitherium crassum* and *Magericyon anceps*) are plotted in a ternary diagram. Prime refers to prime adults. Infant and juvenile categories are lumped together under the juvenile label (Modified from Domingo et al. [25]).
(PDF)

Figure S5 **Number of Identified Specimens (NISP) in the ‘Carnivora indet.’ and ‘Indet.’ taxonomic categories in BAT-1 LLA.** Abbreviations as in Figure 2.
(PDF)

Figure S6 **BAT-1 LLA excavation grid showing the stereographic projections per each of the squares.** Red arrows correspond to the mean trend exhibited by the bones and is shown in those squares where the fossil material had a preferred orientation.
(PDF)

Figure S7 **Spatial distribution of BAT-1 LLA fossils in a SE-NW vertical section.** Each point corresponds to a fossil (the XYZ coordinates were measured in the middle part of each bone). Note that the remains were deposited on a surface sloping towards the northwest. Letters on the horizontal axis correspond to the grid names. The vertical axis represents depth (Z coordinate). This graph is based on the material recovered between 2001 and 2008.
(PDF)

Figure S8 **Weathering alteration analysis in BAT-1 LLA remains.** A, Weathering stages (WS) exhibited by the bones. Raw NISPs are indicated above the bars; B, Metacarpal III (B-1619) of *Machairodus aphanistus* exhibiting a WS 0 ( = intact bone), most of the bones from BAT-1 LLA display this weathering stage; C, Hemimandible (B-3358) of *Protictitherium crassum* exhibiting a WS 1; D, Undetermined bone fragment (B-3314) displaying a WS 2.
(PDF)

Figure S9 **Bone breakage in BAT-1 LLA.** A, Degree of bone completeness (all bones); B, Angle of fracture (only long bones). Perp. = perpendicular, longit. = longitudinal; C, Type of fracture surface (only long bones). Irregul. = irregular. Raw NISPs are indicated above the bars. ‘Other’, in (B) and (C), refers to other fracture combinations too scarce to plot them separately; D, Rib showing multiple perpendicular smooth fractures.
(PDF)

Figure S10 **Skeletal element proportions of** ***Hipparion*** **sp. expressed as Relative Abundance (%Ri) in BAT-1 ULA.** Transport groups are given in roman numerals following Voorhies [29] and Behrensmeyer [30]. Group I: inmediately transported elements, Group II: elements transported gradually, Group III: lag deposit. Group I-II is an intermediate category. Abbreviations as in Figure 2.
(PDF)

Figure S11 **Bone breakage in BAT-1 ULA.** A, Degree of bone completeness (all bones). Indet. = indetermined, fossils for which it was not possible to determined their degree of completeness; B, Angle of fracture (only long bones). Perp. = perpendicular, longit. = longitudinal; C, Type of fracture surface (only long bones). Irregul. = irregular. ‘Other’ refers to other fracture combinations too scarce to plot them separately. Raw NISPs are indicated above the bars.
(PDF)

Table S1 **Skeletal completeness for BAT-1 ULA well-defined taxa.** All the elements were used in the estimation of the skeletal completeness.
(XLS)

Text S1 **Supporting information text.**
(DOC)
