# Supplementary figures and images for: Origin of an Assemblage Massively Dominated by Carnivorans from the Miocene of Spain
Source: PLoS One. 2013 May 1;8(5):e63046. doi: 10.1371/journal.pone.0063046 (PMC3641116; doi:10.1371/journal.pone.0063046)

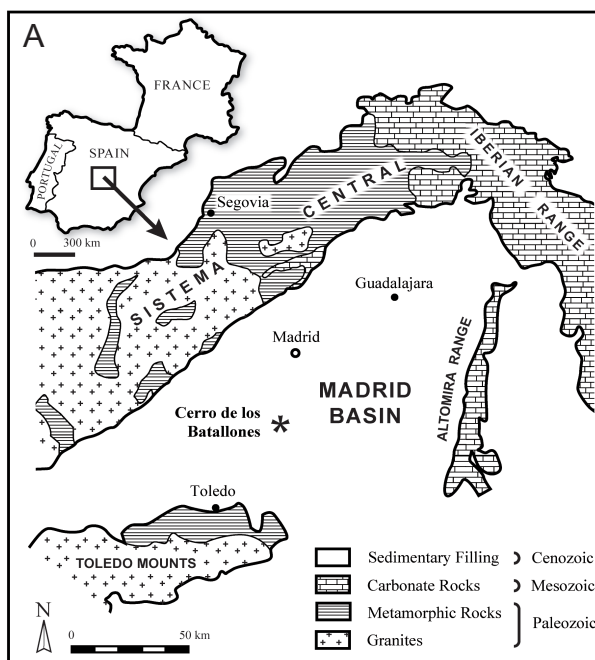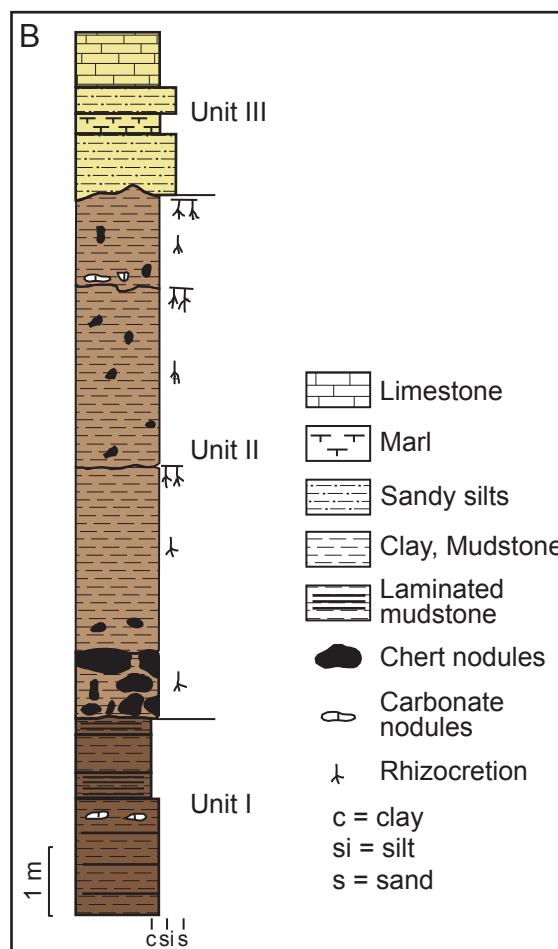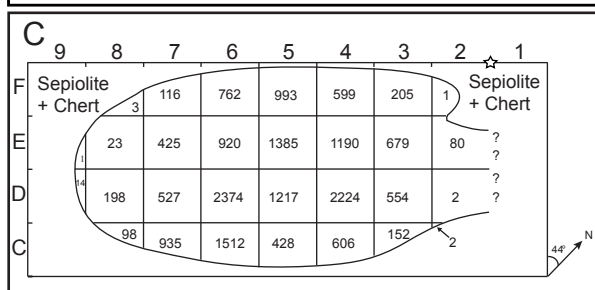

Supplement: Figure S1 — Cerro de los Batallones and Batallones-1 geologic and taphonomic context. A, Location of Cerro de los Batallones within the Madrid Basin (Modified from Calvo et al. [18]); B, Stratigraphic column of Cerro de los Batallones (Modified from Morales et al. [6]). Location of the fossil bones is not indicated since they are embedded in a sedimentary unit that discordantly cuts the three main units of the butte; C, Plant view of the grid system used in the excavation of Batallones-1. Each of the grids is named after a letter and a number (indicated on the border of the drawing) and has 2×2 m dimensions. Numbers within each of the grids is the total amount of large mammal fossils recovered throughout all the field seasons (1991–1993 and 2001–2008). The star corresponds to the (0, 0) coordinate for the X and Y of the system used to locate the fossils. Question marks indicate that the limits of the Batallones-1 LLA have not been found in that area of the fossil site. (PDF) [file pone.0063046.s001.pdf]

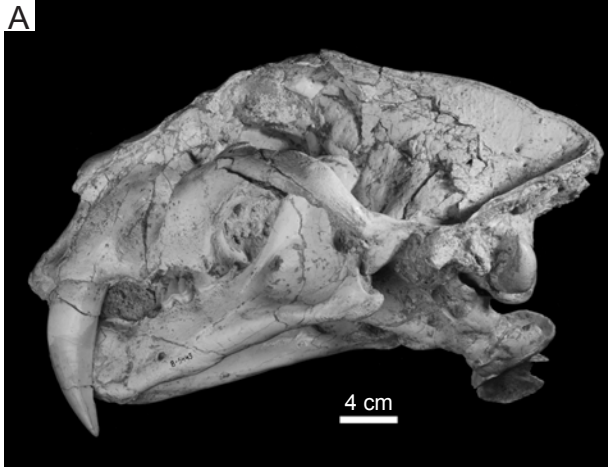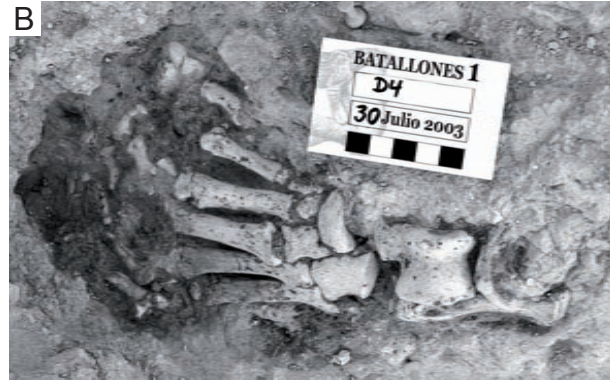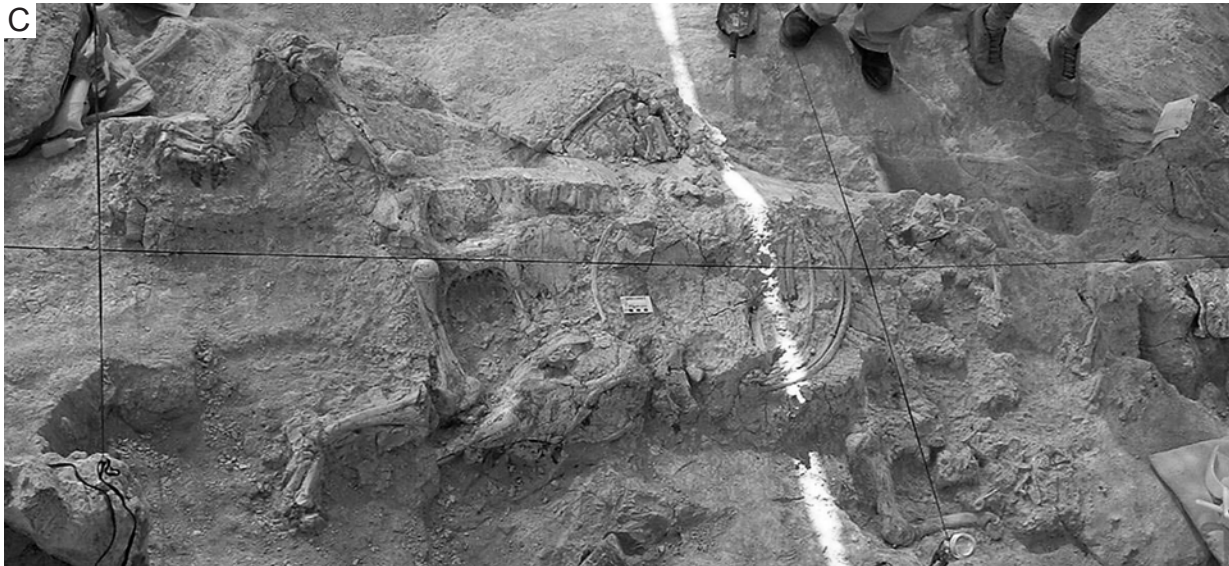

Supplement: Figure S2 — Fossils from BAT-1 LLA. A, Skull and mandible of the sabertoothed cat Machairodus aphanistus (B-5445); B, Articulated foot of the amphicyonid Magericyon anceps. The scale represents 5 cm; C, Articulated skeleton of the rhinoceros Aceratherium incisivum. Note that the skull is disarticulated and displaced. (PDF) [file pone.0063046.s002.pdf]

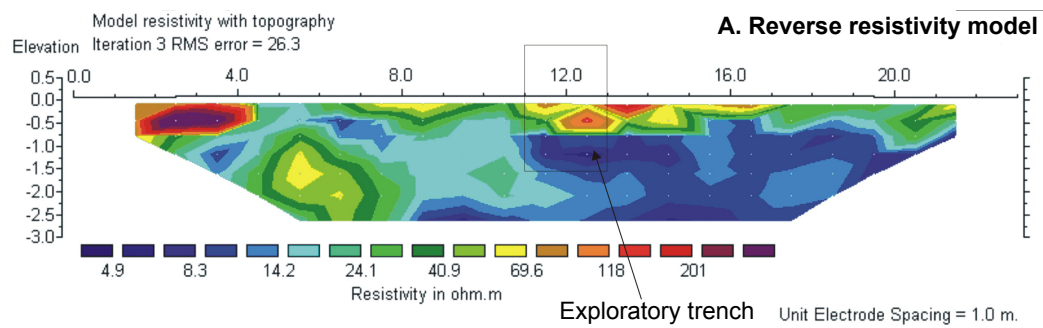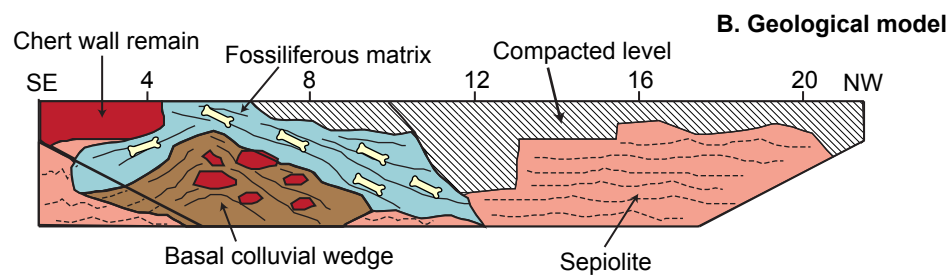

Supplement: Figure S3 — Geophysical profile of BAT-1 LLA. A, Electrical resistivity tomography 2D inverse model performed parallel to the northeast wall of BAT-1 LLA (vertical section) (Modified from Morales et al. [6]). The fossiliferous content of the fossil site was prospected in the 1991–1993 period in the location marked as ‘exploratory trench’. The arrangement of the electrodes is given in the horizontal axis. The electrodes were 1 meter away from each other. The depth of the survey (in m) is provided in the vertical section; B, Geological interpretation of the geophysical model. (PDF) [file pone.0063046.s003.pdf]

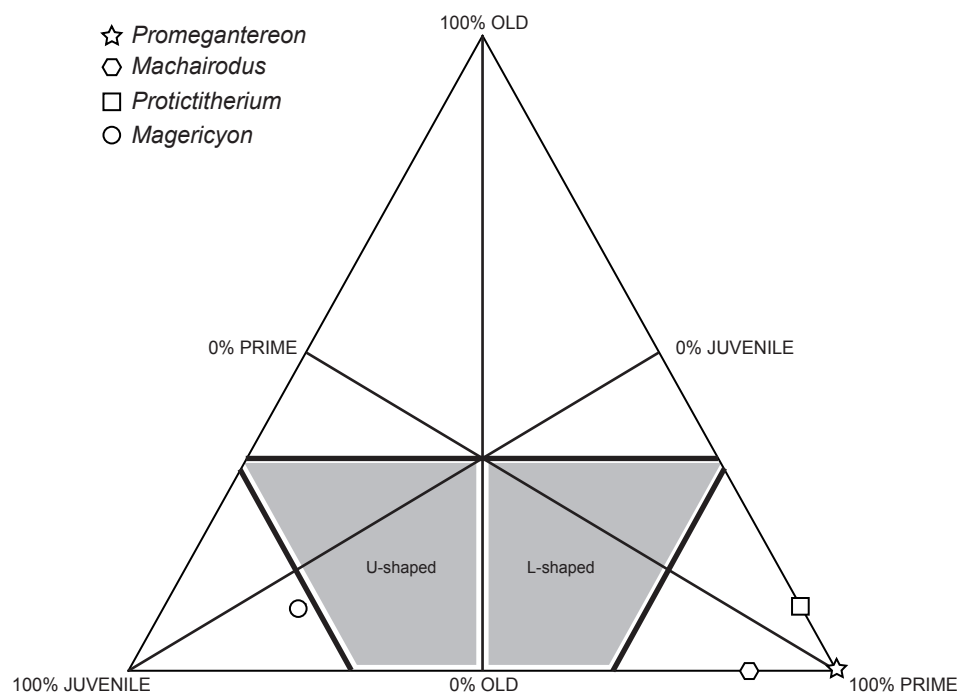

Supplement: Figure S4 — Age structure of the four most common taxa from Batallones-1 LLA. Age structure of the taxa analyzed (Promegantereon ogygia, Machairodus aphanistus, Protictitherium crassum and Magericyon anceps) are plotted in a ternary diagram. Prime refers to prime adults. Infant and juvenile categories are lumped together under the juvenile label (Modified from Domingo et al. [25]). (PDF) [file pone.0063046.s004.pdf]

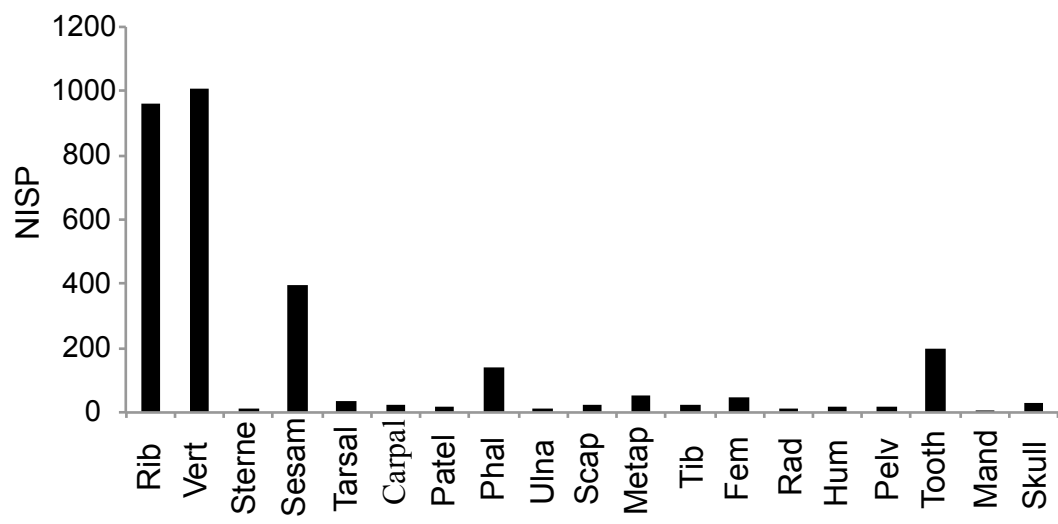

Supplement: Figure S5 — Number of Identified Specimens (NISP) in the ‘Carnivora indet.’ and ‘Indet.’ taxonomic categories in BAT-1 LLA. Abbreviations as in Figure 2. (PDF) [file pone.0063046.s005.pdf]

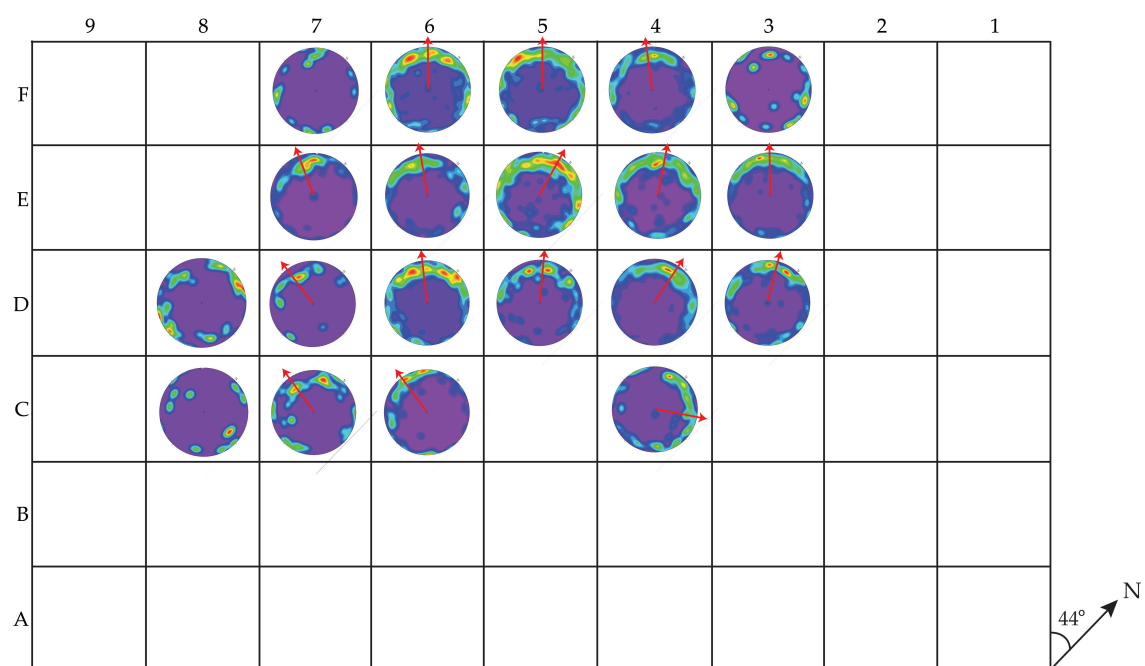

Supplement: Figure S6 — BAT-1 LLA excavation grid showing the stereographic projections per each of the squares. Red arrows correspond to the mean trend exhibited by the bones and is shown in those squares where the fossil material had a preferred orientation. (PDF) [file pone.0063046.s006.pdf]

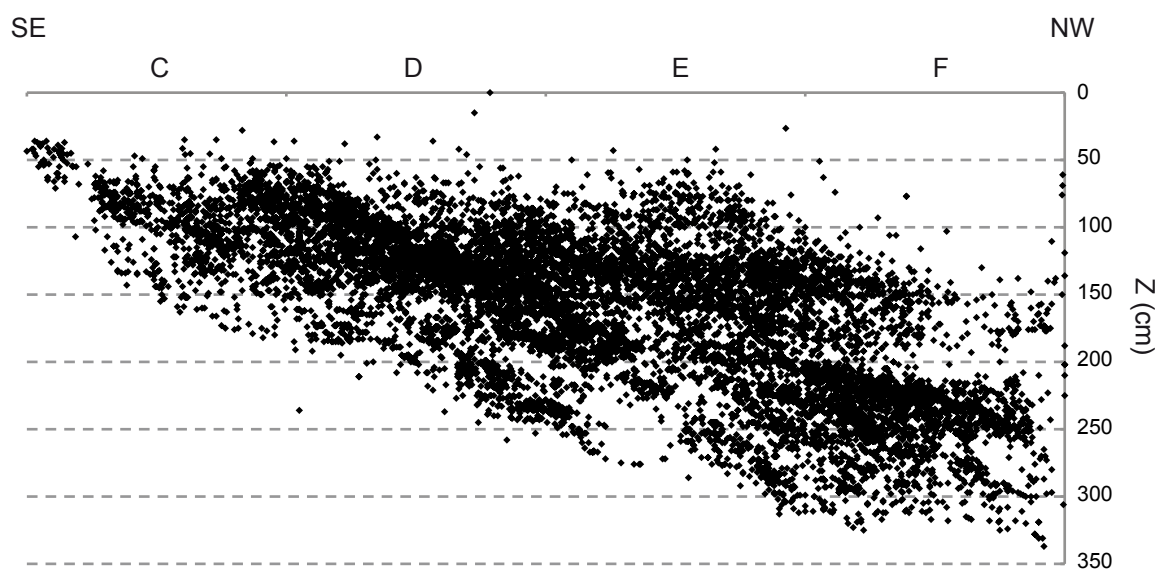

Supplement: Figure S7 — Spatial distribution of BAT-1 LLA fossils in a SE-NW vertical section. Each point corresponds to a fossil (the XYZ coordinates were measured in the middle part of each bone). Note that the remains were deposited on a surface sloping towards the northwest. Letters on the horizontal axis correspond to the grid names. The vertical axis represents depth (Z coordinate). This graph is based on the material recovered between 2001 and 2008. (PDF) [file pone.0063046.s007.pdf]

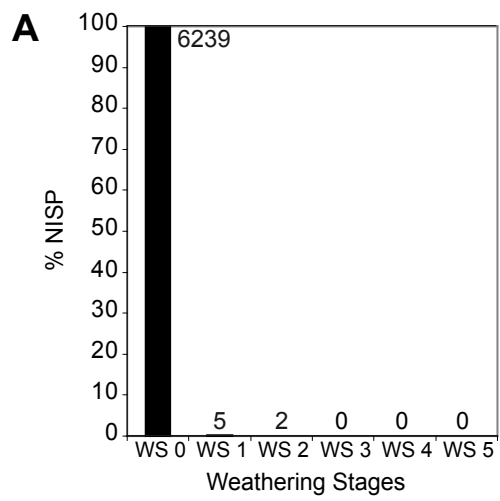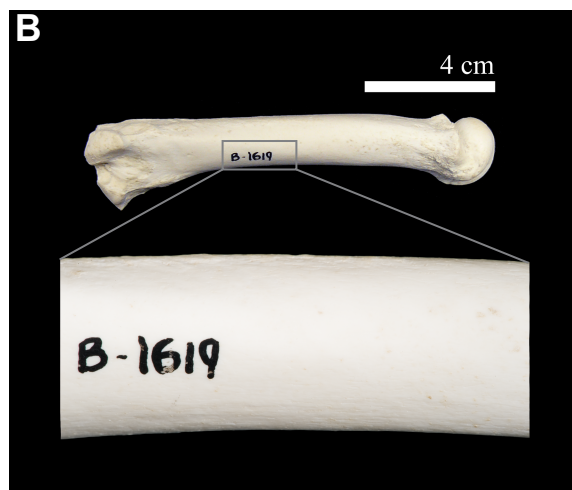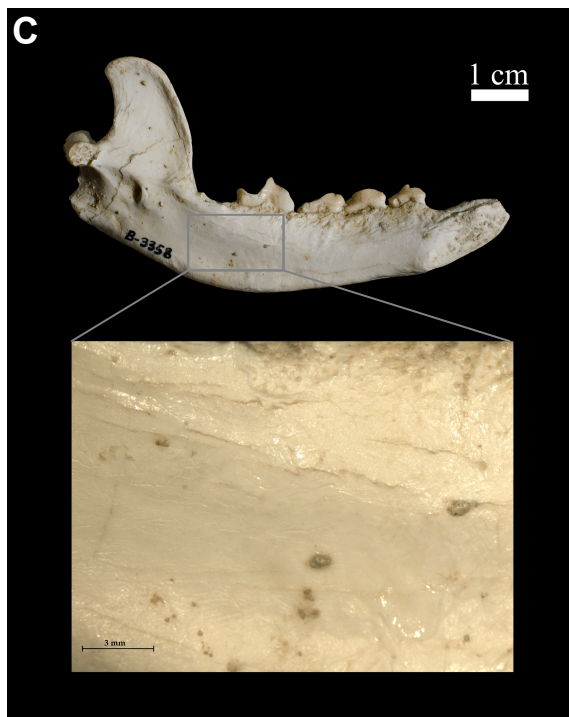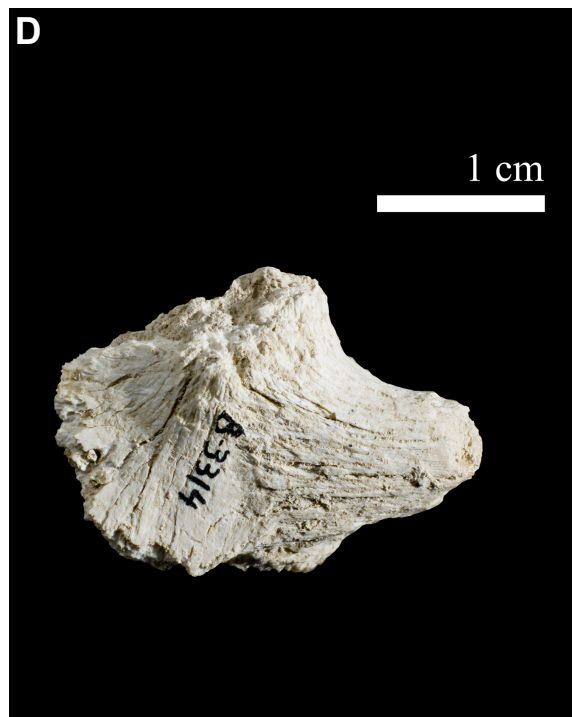

Supplement: Figure S8 — Weathering alteration analysis in BAT-1 LLA remains. A, Weathering stages (WS) exhibited by the bones. Raw NISPs are indicated above the bars; B, Metacarpal III (B-1619) of Machairodus aphanistus exhibiting a WS 0 ( = intact bone), most of the bones from BAT-1 LLA display this weathering stage; C, Hemimandible (B-3358) of Protictitherium crassum exhibiting a WS 1; D, Undetermined bone fragment (B-3314) displaying a WS 2. (PDF) [file pone.0063046.s008.pdf]

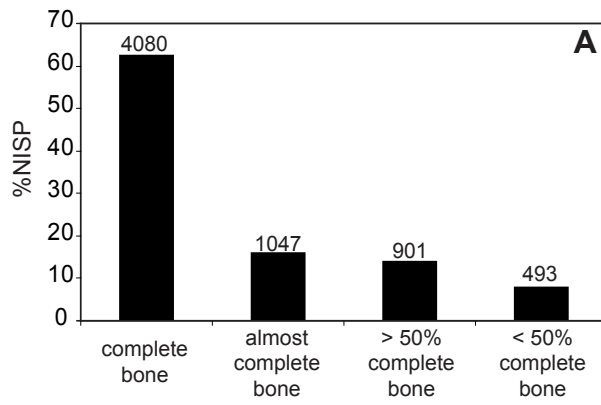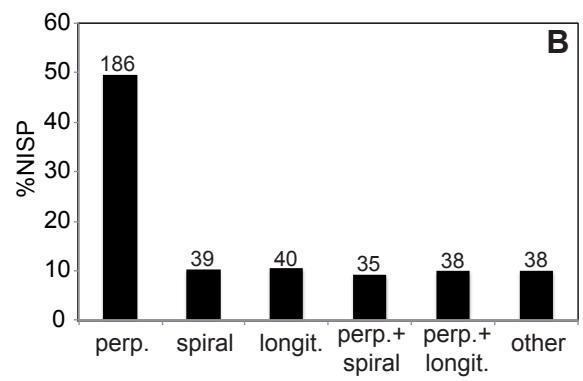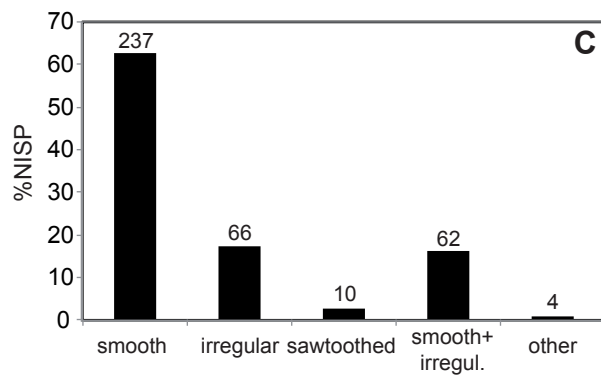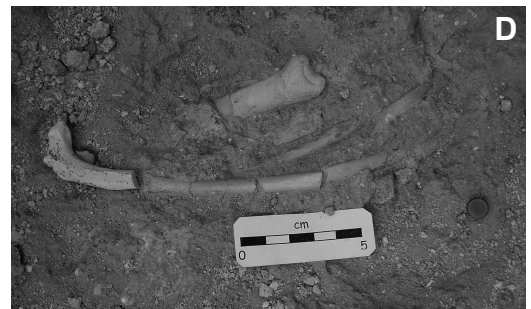

Supplement: Figure S9 — Bone breakage in BAT-1 LLA. A, Degree of bone completeness (all bones); B, Angle of fracture (only long bones). Perp. = perpendicular, longit. = longitudinal; C, Type of fracture surface (only long bones). Irregul. = irregular. Raw NISPs are indicated above the bars. ‘Other’, in (B) and (C), refers to other fracture combinations too scarce to plot them separately; D, Rib showing multiple perpendicular smooth fractures. (PDF) [file pone.0063046.s009.pdf]

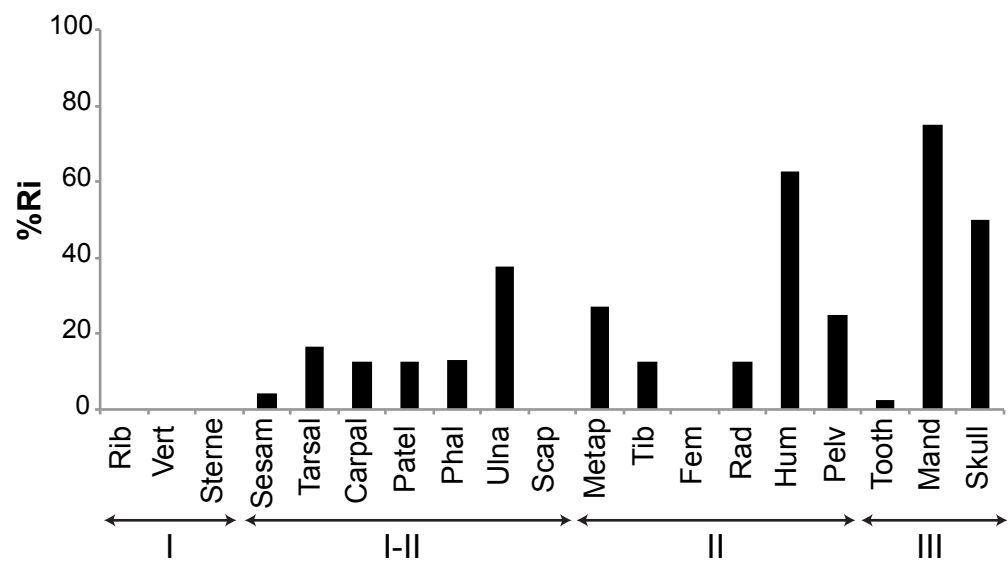

Supplement: Figure S10 — Skeletal element proportions of Hipparion sp. expressed as Relative Abundance (%Ri) in BAT-1 ULA. Transport groups are given in roman numerals following Voorhies [29] and Behrensmeyer [30]. Group I: inmediately transported elements, Group II: elements transported gradually, Group III: lag deposit. Group I-II is an intermediate category. Abbreviations as in Figure 2. (PDF) [file pone.0063046.s010.pdf]

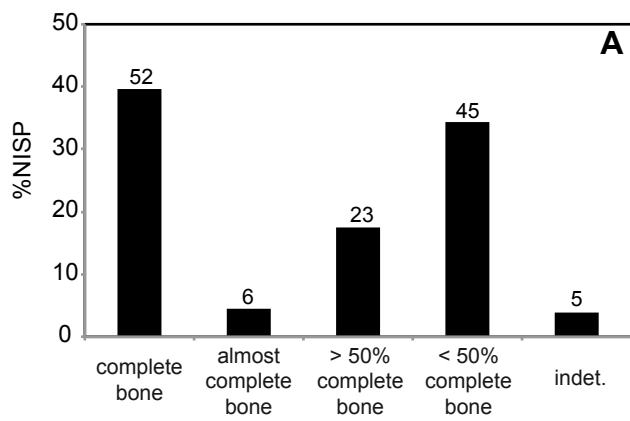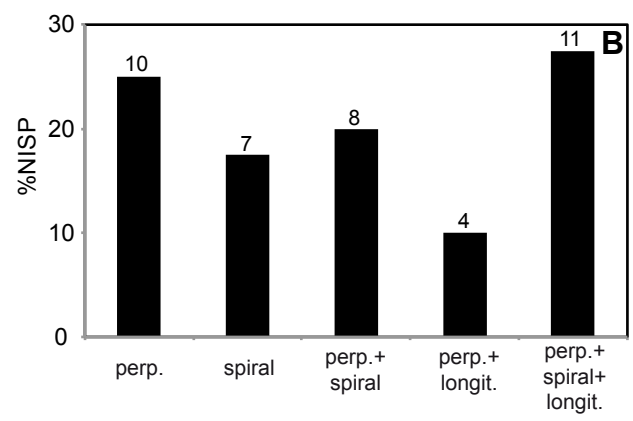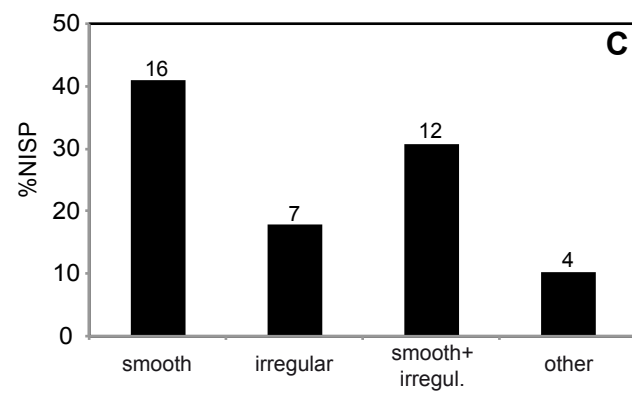

Supplement: Figure S11 — Bone breakage in BAT-1 ULA. A, Degree of bone completeness (all bones). Indet. = indetermined, fossils for which it was not possible to determined their degree of completeness; B, Angle of fracture (only long bones). Perp. = perpendicular, longit. = longitudinal; C, Type of fracture surface (only long bones). Irregul. = irregular. ‘Other’ refers to other fracture combinations too scarce to plot them separately. Raw NISPs are indicated above the bars. (PDF) [file pone.0063046.s011.pdf]
