# Supplementary material for: Origin of an Assemblage Massively Dominated by Carnivorans from the Miocene of Spain
Source: PLoS One. 2013 May 1;8(5):e63046. doi: 10.1371/journal.pone.0063046 (PMC3641116; doi:10.1371/journal.pone.0063046)
Supplement: Text S1 — Supporting information text. (DOC) [file pone.0063046.s013.doc]

**Supporting information text for**

**Origin of an assemblage massively dominated by carnivorans from the Miocene of Spain**

M. Soledad Domingo^1*^, M. Teresa Alberdi^2^, Beatriz Azanza^3^, Pablo G. Silva^4^ and Jorge Morales^2^

^1^*Museum of Paleontology, University of Michigan, Ann Arbor, Michigan, United States of America*

^2^*Departamento de Paleobiología, Museo Nacional de Ciencias Naturales-CSIC, Madrid, Spain*

^3^*Departamento de Ciencias de la Tierra, IUCA, Facultad Ciencias, Universidad de Zaragoza, Zaragoza, Spain*

^4^*Departamento de Geología, Universidad de Salamanca, Escuela Politécnica Superior de Ávila, Ávila, Spain*

^*^Corresponding author. Tel.: +1 734 255 8936

*E-mail address*: soldomingo@gmail.com

**Detailed description of BAT-1 LLA geophysical profile.**

In 2002, a geophysical survey was carried out in BAT-1 to further investigate the topography of the chamber. The progression of the excavations from 2002 to 2008 allowed us to further delineate the geometry of the cave and to refine the interpretations preliminarily attained from the geophysical survey. The profile was performed parallel to the northeast wall (N140ºE) and consisted of 24 electrodes 1 meter away from each other (Fig. S3) [1]. Electrodes 4 to 12 covered the excavated area. The reverse resistivity model revealed a resistive unit to the east that testified the 6 m-thick opal level that had to be removed in the course of the excavations (Fig. S3). This level disappeared at a depth of 0.6-0.9 m of the geophysical profile. Adjacent to this resistive unit, a structure with a bell-shaped geometry was recognized. The resistivity of this structure decreased from its center outwards in a quasi-radial triangular pattern. This bell-shaped unit corresponded to a basal colluvial breccia in which large-sized boulders (0.5-0.7 m in diameter) of sepiolite, limestone and opal are embedded in a fine-grained marly siliciclastic matrix with a variable content of clay intraclasts. These boulders corresponded to fallen blocks coming from the wall or ceiling of the cavity. Adapting to the triangular structure, there were less resistive materials. The course of the excavations revealed that these materials corresponded to the fossil bearing beds. These materials had a preferential leaning towards the W and NW due to the larger extension of this flank of the colluvial wedge and the reduced space that existed on the east side of the site due to the presence of the opal wall that prevented the development of the other flank of the wedge (Fig. S3). Between electrodes 11-24, the geophysical investigation revealed the presence of a pseudohorizontal upper level that corresponded to the occurrence of artificial fillings and soil compacted by the transit of machinery and people. Beneath this level, there was a less resistive material that corresponded to the sepiolite substratum (Unit II).

**Detailed Materials and Methods**

During the excavation, we identified the remains with a sample number and determined their taxonomical and anatomical adscription (these data were double-checked and refined during the preparation and taphonomic evaluation of the remains), their location in a XYZ coordinate system and their trend and plunge (in bones with longer axis).

BAT-1 excavation took place in two periods: the first one, between 1991 and 1993, and the second one, between 2001 and 2008. The enormous number of remains collected made it unfeasible to carry out taphonomical observations on all of them. The selection of a subsample was necessary. The material collected between 1991 and 1993 was considered to be an appropriate sample since almost the entire collection was prepared by the time our taphonomical investigation began; therefore, this sample was potentially not affected by bias coming from preferential preparation of remains with a particularly high taxonomical interest. A total of 6,739 bones were included in our taphonomical study (this is ~35% of the total large mammal remains found throughout all the field seasons). Of this subsample, only 142 fossils belong to BAT-1 ULA since this level was almost completely destroyed during the sepiolite extraction. Only 80 remains from BAT-1 ULA were found *in situ* and, therefore, had XYZ coordinates. The remaining BAT-1 ULA bones were extracted by the mining machinery and lacked XYZ coordinates. Therefore, results provided in this study cannot be considered as definitive and complete for this upper assemblage. Taphonomic observation for BAT-1 ULA was carried out in 132 remains. The 10 remaining bones (belonging to rhinoceros and proboscidean species and one *Hipparion* sp. remain) were found in the collections after the taphonomic study was concluded and are, therefore, excluded from the bone modification analysis but included in taxonomical and anatomical quantifications.

All the bones examined in this work belong to large mammals of the orders Carnivora, Perissodactyla and Artiodactyla.

Number of Identified Specimens (NISP), Minimum Number of Elements (MNE) and Minimum Number of Individuals (MNI) were calculated following Lyman [2]. Attributes such as taxon, element, side (right or left), portion of the element recovered, age-class, and association-articulation were considered for the MNE determination. Minimum Number of Individuals (MNI) was derived from the most common element for each taxon [3].

To investigate skeletal completeness in BAT-1 LLA, we used information coming from all the field seasons (not just the subsample) as it provides a more complete picture of the individuals' representation in the assemblage. A cautionary note must be pointed out: while all the material coming from the 1991-1993 was directly examined, estimates on the number of remains recovered during 2001-2008 seasons are based on the field notes. The implication is that, while 1991-1993 NISP could be reduced to MNE, reduction of NISP to MNE was not possible for the 2001-2008 material because field notes did not sistematically recorded the exact proportion of the recovered remain, side or age-class. MNE is an index preferred to NISP because it avoids the overestimation of elements that have undergone breakage [2]. However, in BAT-1 LLA, NISP and MNE are highly correlated (Spearman's D = 0.5; p (uncorrelated) = 0.003) because fragmentation of remains is low. Accordingly, we believe that BAT-1 LLA NISP can be considered a good index to assess the representation of the remains recovered between 2001 and 2008 until a future direct examination of this sample allows us to estimate the MNE.

The analysis of skeletal completeness was conducted only on those taxa identified to the species level by the time our taphonomical investigation was underway and on monospecific taxa. The completeness was estimated after excluding from the quantifications teeth (only in the case of the carnivoran taxa) and several other skeletal elements. Most of the teeth of the carnivoran from BAT-1 LLA are retained in mandibles and maxillae so counting them as separate skeletal elements would result in an underestimation of the completeness of these species. In turn, because skulls and mandibles were not common among the herbivores, we did include isolated teeth in the completeness analysis for these taxa. Vertebrae, ribs, sesamoids (other than patella) and sternebrae are difficult to assign with confidence to a particular species because they lack resolutive characters. Consequently, it is possible that they can be mistakenly assigned to a particular species or left as an element without a taxonomical adscription (either Carnivora indet. or Indet.). To avoid underestimations of the completeness percentage, we removed these elements from our completeness estimate. The formula used to gauge the skeletal completeness is the same one used to calculate Relative Abundance (see below, [4]) but utilizing the total number of observed and expected elements per taxa (i.e., the completeness of whole skeletons is assessed instead of counting the proportions of particular elements). To evaluate whether there are biases or not in the skeletal representation of the different taxa (i.e., whether carcasses were deposited complete or not), we performed Kolmogorov-Smirnov tests comparing the observed skeletal representation and the expected skeletal representation (for the MNI estimated for each taxa). In the statistical analysis, we excluded from the expected values the teeth (only in the case of carnivorans) and the vertebrae, ribs, sesamoids and sternebrae to allow comparability with the observed values. Significance for this test was established at the p = 0.01 level. We did not perform statistical tests on the skeletal completeness of the BAT-1 ULA because, in this assemblage, the underrepresentation of skeletal elements is most likely due to the destruction of most of the concentration and this fact biases the evaluation of whether or not skeletons were deposited complete.

Relative abundance (Ri) was calculated to depict the representation of specific skeletal elements for each of the analyzed taxa. Relative abundance was calculated using the formula established by Andrews [4]:

R_i_ = MNE_i_ / (MNI × E_i_) × 100

where R_i_ is the relative abundance of element i, MNE_i_ is the minimum number of element i in the sample, MNI is the minimum number of individuals in the taxon of interest, and E_i_ is the number of times that element i appears in a complete skeleton.

The susceptibility of the specimens to transport was investigated by the quantification of elements belonging to the transport groups defined by Voorhies [5]. Voorhies [5] conducted flume experiments using disarticulated sheep and coyote skeletons and recognized three hydraulic-transport groups (with two transitional groups) that were later complemented by Behrensmeyer [6]. Group I contains the most easily transported elements while Group III is made up of elements less prone to be transported (lag deposit). We classified remains according to Voorhies categories to evaluate whether the assemblage was subject to transport. The possible influence of transport on an assemblage can also be detected through the analysis of the orientation of bones and the quantification of abraded remains.

The analysis of the XYZ coordinates of the remains can also help to clarify the geometry of the bone assemblage and shed light on the two- and three-dimensional distribution of skeletal remains [7]. Spatial data were directly collected during the field seasons so information coming from the two excavation periods was available. The 1991-1993 Z reference point, that measured the depth at which fossils were found, was lost by the beginning of the second excavation period in 2001. This fact hinders the joint analysis of data coming from the two periods of excavation. We performed separate spatial analysis for each period and they yielded very similar results. In this study, we present the spatial data from the 2001-2008 period because it contains a larger number of remains.

Bone alteration caused by weathering, abrasion, trampling, carnivoran and rodent activity, root action and fragmentation was analyzed. Based on the study of carcasses from Amboseli National Park, Behrensmeyer [8] defined six weathering stages from intact bone (Stage 0) to extremely weathered bone (Stage 5).

Abrasion was measured in three stages: intact (0), moderately rounded bone (1) and extremely polished bone (2) [9]. Carnivoran tooth marks were classified as pits (shallow oval depressions), punctures (deep oval depressions), scores (elongate marks) and furrows (contiguous grooves generally found at the ends of long bones) [2].

Bone breakage intensity was analyzed by determining the integrity of each identifiable element using the next categories: complete, almost complete (bones only missing some bone flakes), more than one-half complete and one-half or less complete. Besides, we recorded the fracture angle relative to the long axis of the specimen (perpendicular, spiral or longitudinal) and the morphology of the fracture surface (smooth, irregular, sawtoothed, V-shaped, columnar and flaking) [2]. These two last fracture variables were analyzed on long bones, which included the humerus, radius, ulna, femur, tibia, fibula and metapodials (except for the metacarpal I and metatarsal I of the felids that are short bones). We excluded the phalanges because they are shorter, smaller and more compact elements so their response to breakage might be different from that of longer bones.

**Results**

LLA Assemblage Data

When we analyze the skeletal representation in the taxonomical categories 'Carnivora indet.' and 'Indet.', we can see that the number of vertebrae, ribs and sesamoids is high compared to their values in the particular taxa (Fig. S5). As previously mentioned, this is due to the difficulties in taxonomically identifying these elements. Besides, the estimates for vertebrae and ribs must be considered a minimum approximation because: 1) some articulated spines were found during the excavations but their vertebrae were not counted as separated elements but assigned a single sample number in the 2001-2008 field notes and 2) it is probable that some vertebrae and ribs from the 1991-1993 field season remain non-prepared and were not counted here.

**Discussion**

Further information on the patterns of accumulation in BAT-1 LLA

To determine the concentration mode for the Felinae indet. and Mustelidae/Mephitidae indet. fossils, it will be necessary to wait until their remains are identified to the species level.

As for the concentration mode of the herbivores, the analysis of the completeness of the suid *Microstonyx* sp. indicates that the complete skeleton of the only individual represented in BAT-1 LLA was deposited (Table 3). It is likely that this individual accidentally fell in the cave. The *Microstonyx* sp. individual is a juvenile so it might be possible that some elements were destroyed due to their lower density. The equid *Hipparion* sp. is represented by two incomplete individuals. The taphonomical features exhibited by the *Hipparion* sp. bones, including their Rare Earth Element patterns, are similar to those observed in the rest of the assemblage [10] so they seem to have undergone a similar taphonomical history than the rest of the remains. It is probable that some skeletal parts remain unexcavated. In any event, the reason behind the underrepresentation of *Hipparion* sp. skeletal elements is still unknown.

Different characteristics observed in the musk deer remains imply that these individuals were preyed or scavenged outside the cavity and eventually incorporated to the assemblage in their predator/scavenger digestive system when it became trapped [11]. Although it was not possible to assess the completeness of the rhinoceroses from BAT-1 LLA, the completely articulated skeleton of an *Aceratherium incisivum* individual implies that it fell into the cave probably as a consequence of an accident (Fig. S2C).

Detailed information on the breakage of remains in BAT-1 ULA

The amount of broken bones is significantly larger in the ULA than in the LLA, both if the total sample is considered or if only the long bones are analyzed (Table 1, Figs. S9 and S11). It is difficult to determine the process behind the breakage of bones in the ULA. Hydraulic transport, carnivore activity and trampling do not seem to have largely affected ULA remains (Table 1). Weathering and root activity are processes that altered more bones from the ULA than the LLA so they might have fractured some of the bones. The amount of perpendicular breakages in long bones are less common in this level than in the LLA which would be indicating that the sediment compaction was not a major fragmentation agent in the ULA. This is coherent with the smaller weight of sediment that ULA bones withstood compared to LLA bones.

**References**

1. Morales J, Pozo M, Silva PG, Domingo MS, López-Antoñanzas R, et al. (2008) El sistema de yacimientos de mamíferos miocenos del Cerro de los Batallones, Cuenca de Madrid: estado actual y perspectivas. Paleontologica Nova SEPAZ 8: 41-117.

2. Lyman RL (1994) Vertebrate Taphonomy. Cambridge: Cambridge University Press. 524 p.

3. Shipman P (1981) Life history of a fossil. An introduction to Taphonomy and Paleoecology. Cambridge: Harvard University Press. 222 p.

4. Andrews P (1990) Owls, Caves and Fossils. Chicago: The University of Chicago Press. 239 p.

5. Voorhies MR (1969) Taphonomy and population dynamics of an early Pliocene vertebrate fauna, Knox County, Nebraska. Contributions to Geology, University of Wyoming Special Paper 1: 1-69.

6. Behrensmeyer AK (1975) The taphonomy and paleoecology of Plio-Pleistocene vertebrate assemblages East of Lake Rudolph, Kenya. Bulletin of the Museum of Comparative Zoology 146: 473-578.

7. Eberth DA, Rogers RR, Fiorillo AR (2007) A practical approach to the study of bonebeds. In: Rogers RR, Eberth DA, Fiorillo AR, editors. Bonebeds: Genesis, Analysis, and Paleobiological Significance. Chicago: The University of Chicago Press. pp 265-331.

8. Behrensmeyer AK (1978) Taphonomic and ecologic information from bone weathering. Paleobiology 4: 150-162.

9. Alcalá L (1994) Macromamíferos neógenos de la fosa de Alfambra-Teruel. Teruel: Instituto de Estudios Turolenses. 554 p.

10. Domingo MS, Domingo L, Sánchez IM, Alberdi MT, Azanza B, Morales J (2011) New insights on the taphonomy of the exceptional mammalian fossil sites of Cerro de los Batallones (Late Miocene, Spain) based on Rare Earth Element geochemistry. Palaios 26: 55-65.

11. Domingo MS, Sánchez IM, Alberdi MT, Azanza B, Morales J (2012a) Evidence of predation/scavenging on Moschidae (Mammalia, Ruminantia) from the Late Miocene of Spain. Lethaia 45: 386-400.
